# Cancer registries in Pakistan: a scoping review

**DOI:** 10.1016/j.lansea.2025.100615

**Published:** 2025-06-14

**Authors:** Sehar Salim Virani, Kaleem Sohail Ahmed, Megan Springer, Muzamil Hussain, Leslie Christensen, Farah Asif, Shahid Pervez, Zehra Fadoo, Asim Belgaumi, Syed Nabeel Zafar

**Affiliations:** a Department of Surgery, Aga Khan University, Karachi, Pakistan; b Department of Surgery, University of Wisconsin – Madison, Madison, WI, USA; c University of Wisconsin School of Medicine and Public Health, Madison, WI, USA; d Medical College, Aga Khan University, Karachi, Pakistan; e Ebling Library for the Health Sciences, University of Wisconsin School of Medicine and Public Health, Madison, WI, USA; f Department of Clinical Research, Shaukat Khanum Memorial Cancer Hospital and Research Center, Lahore, Pakistan; g Department of Pathology and Laboratory Medicine, Aga Khan University, Karachi, Pakistan; h Department of Oncology, Aga Khan University, Karachi, Pakistan; i University of Wisconsin Carbone Cancer Center, Madison, WI, USA

**Keywords:** Cancer, Registry, Pakistan, Scoping review, Cancer registry

## Abstract

Cancer incidence is increasing globally. Although Pakistan does not have a unified national cancer registry, several institutional and regional cancer registries can provide vital information for cancer planning. Following the Joanna Briggs Institute and PRISMA-ScR guidelines, we conducted a comprehensive search across multiple databases and grey literature. Data were extracted regarding registry characteristics, data collection methods, and study details, and findings were summarised narratively to highlight key attributes and data gaps. Of 3714 unique abstracts screened, 102 studies met inclusion criteria, including 92 reporting registry data and 10 describing registry characteristics without patient-level data. Seventeen cancer registries were identified, with varying scope and geographical coverage. Only 19 of Pakistan's 129 cities contribute data to at least one registry. Data collection methods ranged from paper-based forms to advanced software systems. The Karachi Cancer Registry was noted for its high research output. Funding sources were limited, and several registries faced operational challenges. This Review highlights Pakistan's fragmented cancer registry landscape. While important policy-level data can be obtained from existing registries, there is an urgent need for strategic efforts and stakeholder collaboration to establish a national cancer registry system. Such a system could enhance cancer surveillance, inform public health efforts, and serve as a model for similar initiatives in south and southeast Asia.

## Introduction

Cancer registries play a crucial role in providing population-based data on cancer trends and treatment efficacy, enabling health authorities to monitor disease patterns, guide policy-making, and allocate resources effectively.^1^ However, many low- and middle-income countries (LMICs) continue to struggle with population-based registration (PBR).^2^

Pakistan, with a population exceeding 241 million,^3^ faces a substantial cancer burden, with an estimated 185,748 new cancer cases and over 118,631 cancer-related deaths in 2022.^4^ Unfortunately, cancer data in Pakistan remains fragmented and inconsistent, primarily due to the absence of a national cancer registry and the reliance on hospital-level data, which often lack population-based representativeness. Efforts to establish a national cancer registry have encountered significant challenges, including limited resources, insufficient funding and infrastructure, lack of government initiatives, and logistical hurdles.^5^ The Ministry of National Health Services Regulations and Coordination designated the Health Research Institute (HRI) to establish a national cancer registry by affiliating major public and private hospitals. However, this initiative has struggled to achieve its goals, with only eight hospitals across Pakistan submitting data on a quarterly basis since May 2015.^6^ While other countries in the southeast Asia region face similar challenges, India has successfully developed a high-quality PBR. India's National Cancer Registry Programme has played a crucial role in shaping public health policies, informing nationwide cancer screening guidelines, and supporting the expansion of regional, state, and tertiary cancer care institutions.^7^

Despite Pakistan's socioeconomic and geopolitical challenges, several single- and multi-institutional cancer registries have emerged, playing a vital role in collecting and managing cancer data at local and regional levels.^8^ In the absence of a national registry, these registries could serve as valuable sources of policy-level data. Understanding their capabilities, strengths, and limitations is essential. We aimed to systematically evaluate the current state of cancer registries in Pakistan by assessing their scope, geographical coverage, data quality, strengths, and limitations. This scoping review identifies critical gaps that need to be addressed and provides recommendations for strengthening cancer registration systems in Pakistan.

## Methods

We conducted a scoping review following the Joanna Briggs Institute methodology for scoping reviews,^9^ adhering to the Preferred Reporting Items for Systematic reviews and Meta-Analyses extension for Scoping Reviews (PRISMA-ScR) guidelines (Supplementary Table S1). The protocol was registered on the Open Science Framework on Aug 25, 2023.^10^

### Article screening

Two reviewers (M.S. and M.H.) independently screened titles and abstracts, with relevant studies advancing to full-text review. Discrepancies were resolved through discussion or consultation with a senior reviewer (S.S.V.). Articles meeting inclusion criteria were included.

### Data extraction

Data extraction was conducted in Covidence by two reviewers (M.S. and M.H.) independently. Discrepancies were resolved through discussion or by consulting a third reviewer (S.S.V.). Data were charted using predefined variables (Supplementary Table S3) without thematic coding.

### Data synthesis

Summaries were created for each cancer registry. Some registries, such as Shaukat Khanum Memorial Cancer Hospital and Research Center (SKMCH&RC) registry, operate independently while also contributing to larger registries (e.g., Punjab Cancer Registry [PCR]); both were described separately. Data was collated and presented graphically, in tables, and narratively. As a scoping review, we did not perform quality assessment or pooling of quantitative data, aligning with the goal of mapping existing literature rather than evaluating bias or the strength of evidence.^11^ We also did not perform any patient-level analyses or calculate age-standardised rates consistent with our focus on mapping the available cancer registries in Pakistan.

## Results

**Fig. 1:**
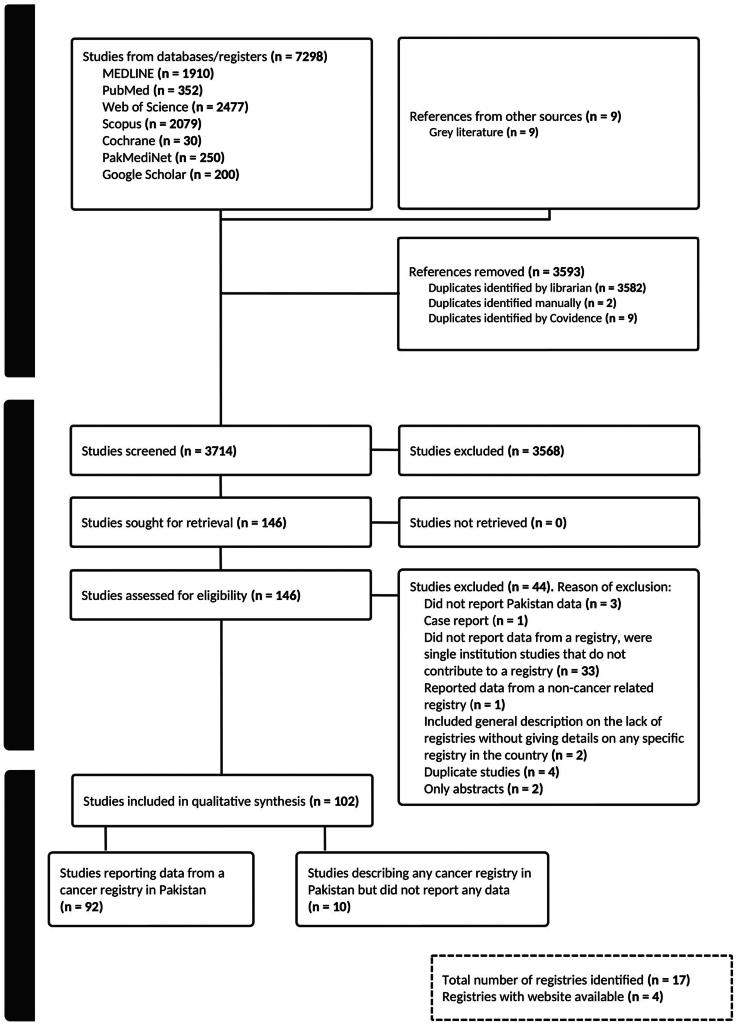
PRISMA flow diagram.

Out of a total of 3714 unique titles and abstracts screened for relevance, 146 studies underwent full-text review. Data from 102 studies were ultimately included in the qualitative synthesis (Fig. 1). Four registries had accessible websites providing additional information, including the Armed Forces Institute of Pathology Rawalpindi Tumor Registry (AFIPR-TR), SKMCH&RC's Cancer Registry, PCR, and Shifa International Hospital Registry (See note below Table 1).

**Table 1:** Summary details of cancer registries in Pakistan.

Registry	Primary responsible institution	Data source	Geographical coverage	Population description	Registry establishment and duration
Armed Forces Institute of Pathology Rawalpindi Tumor Registry	Armed Forces Institute of Pathology, Rawalpindi	Military hospitals and civil, public, and private-sector hospitals from upper Punjab, Khyber Pakhtunkhwa and the adjacent Rawalpindi-Islamabad region	Primarily northern Punjab and Khyber Pakhtunkhwa; samples received from across Pakistan.	Biopsy-proven tumor cases	Established in the 1960s and continues to be active.
National Cancer Registry	Established by the Health Research Institute (HRI), Islamabad and Pakistan Medical Research Council (PMRC)	Not specified	National level	Not specified	1970s–1990s
Shaukat Khanum Memorial Cancer Hospital and Research Centre (SKMCH & RC)'s Hospital Cancer Registry	Shaukat Khanum Memorial Cancer Hospital and Research Center (SKMCH&RC) in Lahore and Peshawar	Shaukat Khanum Memorial Cancer Hospital and Research Center (SKMCH&RC) in Lahore and Peshawar; Karachi Diagnostic Center (KDC)	Contains data from patients all over Pakistan Data shared with the Punjab Cancer Registry and used as a source for IARC (WHO) and ‘Globocan 2018’.		1994 to Present
Karachi Cancer Registry	Established at Sindh Government Services Hospital by the Government of Sindh in collaboration with the International Agency for Research on Cancer (IARC), WHO.	Data was initially collected from over 50 registration sources, including major hospitals, oncology centers, pathology laboratories and death registration offices in both government and private sectors. Post revival, seven tertiary care hospitals of Karachi contribute to the registry.	The registry covers Karachi, with data also including patients presenting from other districts to contributing institutions.	Cancer cases from Karachi seeking diagnosis and treatment.	1995–2012: Initial phase 2017-Present: Revived phase
Punjab Cancer Registry	The registry is managed by a nine-member governing council. At present, the Registry has around 53 members, 9 of whom make up the Governing Council. The Central Office of the Registry is located within the Shaukat Khanum Memorial Cancer Hospital & Research Center, Lahore, Pakistan.	There are 28 participating institutions.	Initially, the registry aimed to collect cancer data from across Punjab. However, starting July 1, 2008, it focused on residents of Lahore. By 2014, the registry expanded to include Faisalabad, Sheikhupura, Kasur, and Nankana Sahib. It added Sialkot and Narowal in 2016, followed by Okara, Gujrat, Jhelum, Rawalpindi/Islamabad in 2018. More recently, Multan, Mandi Bahauddin, Sargodha, and Chiniot were included, with plans to expand to other districts over time.	Cancer cases presenting at the included centers.	The registry was established in 2005 and is ongoing
Aga Khan University Cancer Registry	Aga Khan University Pathology Department—Pathology based cancer registry. The clinical aspects of the registry are dealt with by AKUH Cancer Committee. Aga Khan University, Department of Anaesthesiology–registry database for perioperative anaesthetic management in head and neck cancer surgery.	84 AKU pathology centers and branches across Pakistan.	National coverage, with data collected from across Pakistan. Pathology based registry shares data to the Karachi Cancer Registry.	Patients from across Pakistan.	Pathology based cancer registry—2009 to Present Aga Khan University, Department of Anaesthesiology–registry database for perioperative anaesthetic management in head and neck cancer surgery—started in 2017 to present.
Liaquat National Hospital and Medical College Hematologic Tumour Registry	Liaquat National Hospital and Medical College	Liaquat National Hospital and Medical College	Karachi, Pakistan	Newly diagnosed patients with haematological cancers presenting at LNH	2012–Present
Dow Cancer Registry	Public-sector diagnostic and reference laboratory of Karachi—the Dow Labs.	Pathology-based cancer registry. Samples are collected from 34 strategically placed collection points throughout the city.	Entire city of Karachi, Pakistan. Contributes to Karachi Cancer registry	Registers patients from all over Karachi, representing all districts of the city (∼17.4 million).	Founded in May 2014, currently ongoing.
Cancer Registry of Institute of Radiotherapy and Nuclear Medicine (IRNUM) Peshawar	Institute of Radiotherapy and Nuclear Medicine (IRNUM), Peshawar	Patients with biopsies treated at Institute of Radiotherapy and Nuclear Medicine (IRNUM) Peshawar	North-West Frontier Province (NWFP); Afghanistan (refugees); northern Pakistan	Biopsy-proven tumor cases	Not specified
Tumor Registry at Jinnah Hospital	Jinnah Hospital, Lahore	Jinnah Hospital, Lahore; associated with Allama Iqbal Medical College	All patients presenting at the hospital.	Histologically confirmed cancer patients	Not specified
Pakistan Atomic Energy Commission's (PAEC) Cancer Registry	1. Institute of Nuclear Medicine and Oncology (INMOL), Lahore 2. Karachi Institute of Radiotherapy and Nuclear Medicine (KIRAN)	PAEC runs 19 cancer service delivery centres termed ‘Atomic Energy Cancer Hospitals’ across the country, including all provinces and regions operated in collaboration with the NM&O (Nuclear Medicine & Oncology) Division of Pakistan Atomic Energy Commission (PAEC).	National coverage, with data collected from all provinces and regions of Pakistan. Also includes patients from Afghanistan origin presenting at included centers.	Cancer patients presenting to any of the cancer service delivery centres.	Not specified.
Tumor registry at Allied Hospital, Faisalabad	Allied Hospital, Faisalabad	Allied Hospital, Faisalabad	Faisalabad, Pakistan	Patients presenting at the Allied Hospital.	Not specified
Central Nervous System Tumour Registry at Combined Military Hospital, Rawalpindi	Combined Military Hospital, Rawalpindi	Combined Military Hospital, Rawalpindi	Rawalpindi, Pakistan	Patients presenting at Combined Military Hospital, Rawalpindi	Not specified
Nishtar Medical University Hospital Multan (NMH) Registry	Nishtar Medical University Hospital, Multan	Nishtar Medical University Hospital.	South Punjab, Pakistan	Patients receiving cancer care at Nishtar Medical University Hospital.	Not specified
Shifa International Hospital, Islamabad (SIH) Registry	Shifa International Hospital, Islamabad	Shifa International Hospital, Islamabad	Islamabad, with a focus on the local population served by the hospital.	Patients receiving care at Shifa International Hospital	Not specified
Punjab Institute of Nuclear Medicine (PINUM) Cancer Registry	Punjab Institute of Nuclear Medicine (PINUM)	Punjab Institute of Nuclear Medicine (PINUM)	Punjab, Pakistan Contributes to the Pakistan Atomic Energy Commission's (PAEC) cancer registry	Patients presenting at Punjab Institute of Nuclear Medicine (PINUM)	Not specified
Patel Hospital Cancer Registry Database	Patel Hospital, Karachi	Patel Hospital, Karachi	Karachi, Pakistan	Cancer patients presenting to Patel Hospital, Karachi	Not specified

Note: The websites of four registries were also accessed for additional information.^12^, ^13^, ^14^, ^15^

### Study characteristics

Since 2000, there has been a significant increase in research related to cancer registries in Pakistan. Prior to this period, literature on cancer registries in the country was sparse. Most studies conducted were cross-sectional prevalence studies (85 studies), primarily exploring cancer epidemiology, incidence trends, and regional variations (Supplementary Table S4).

### Overview of cancer registries

We identified 17 cancer registries across Pakistan, varying in scope, geographical coverage, and population descriptions. These registries receive data from military hospitals, civil hospitals, diagnostic centers, specialised oncology centers, and both public and private sector hospitals. The oldest registry, AFIPR-TR, was initiated in the 1960s and remains active. An attempt to establish a national cancer registry in the 1970s by the Health Research Institute (HRI) and Pakistan Medical Research Council (PMRC) failed due to funding constraints.^16^ Subsequently, several registries were established between 1990 and 2010 (Table 1). Supplementary Table S5 lists the data source institutions for multi-institutional level registries.

### Data collection methods utilised by cancer registries in Pakistan

Only two registries described their data collection methods in detail. PCR, a regional registry, collects data from hospitals across Punjab province via paper-based forms and a mobile data application. Its database is maintained within SKMCH&RC's electronic Hospital Information System (HIS). The Karachi Cancer Registry (KCR), a city-level registry, collects data from hospitals within Karachi using CANREG-3 software for data management, with data validation measures such as histological verification and death certificate-only cases. Inclusion in the KCR registry is voluntary, requiring patient consent. Data validation is ensured through regular re-screening and checks for duplicate registrations. The registry temporarily halted operations in 2012 following the passing of its founding member but was revived in 2017. Additional registry details are provided in Table 2.

**Table 2:** Operational details of cancer registries.

Registry	Data collection methods	Database management	Data validation	Data variables^a^	Additional notes
**National level registries**					
National Cancer Registry	–	–	–	–	Aimed at collecting nationwide data
Pakistan Atomic Energy Commission's (PAEC) Cancer Registry	Initially recorded on soft data sheets, then transitioned to computational techniques.	–	–	year; sex; age; region of residence; stage; site	–
**Regional level registry**					
Punjab Cancer Registry	Paper-based forms, mobile data application, telephone follow-ups.	Data management is overseen by an epidemiologist or biostatistician, medical coders, and 10–13 cancer registry officers.	Duplicate registration checks are conducted using various combinations of patient details, including name, age, gender, phone number, address, and tumor morphology, with data integrated into an Oracle-based Hospital Information System developed in-house and utilizing the IARCcrgTools package.	(1) demographic features: patient's name, age, birth date, gender, computerised national identity number, telephone number, address, and location of visits. (2) clinical features: the diagnosis date, primary tumour site, biopsy site, morphology, behaviour, grade, stage, metastasis, laterality, most valid basis of diagnosis, and procedure-related information (doctor's name and hospital); (3) status at last follow-up, date and place of death.	Managed by epidemiologist/biostatistician, medical coders, 10–13 registry officers
**City level registry**					
Karachi Cancer Registry	Hospital visits, patient interviews, computerized hospital records	CANREG-3 software	Histological verification (H.V.) and death certificate-only (D.C.O.) cases, annual re-screening, bi-annual duplicate checks	hospital patient-number; date of incidence; name; age; sex; address; ethnicity; topography; morphology; grading; staging; date of death/last follow-up	Resumed in 2017 as part of NCR with secretariat at PHRC, JPMC
**Institution-based registries**					
Armed Forces Institute of Pathology Rawalpindi Tumor Registry	Data from patients/doctors, numerical coding on computers, hard copy registers	–	–	Name; age; sex; birthplace; duration of symptoms; site; histology; place of longest stay, lymph node involvement, surgical procedures	Uses ICD-O for tumor coding
Cancer Registry of Institute of Radiotherapy and Nuclear Medicine (IRNUM) Peshawar	Qualified and experienced technical staff collect data using Hospital Management Information System (HMIS) software.	–	–	diagnosis; nuclear medicine; treatment with chemotherapy and radiotherapy; age; sex; year; cancer type; occupation; territory	–
Shaukat Khanum Memorial Cancer Hospital and Research Centre (SKMCH & RC)'s Hospital Cancer Registry	Real-time data from medical records	Oracle-based Hospital Information System	–	sex; age; demographic area; comorbidities; topography; symptoms; morphology; stage; grade, breast feeding; use of oral contraceptives or hormone replacement therapy; menopausal status; weight; height; BMI; family history; hormone receptor status (ER, PR, HER2); survival status, complications; socio-economic status; follow up	Maintained by Cancer Registry and Clinical Data Management unit
Tumor Registry at Jinnah Hospital	Tumor registry forms filled on the first patient visit and updated thereafter. Manual data entry.	–	–	age; sex; duration of symptoms; disease stage at presentation	–
Aga Khan University Pathology-based Cancer Registry and Anesthesia—Head and Neck Cancer Registry	Specific cancer registration number assigned. Data updated with each patient revisit. Two main core processes including case finding and abstraction.	US-based software titled CNExT.	Manual and computerized validity checks. Internal and external quality checks are used.	age; sex; name; address; telephone numbers; nature of surgery; medical registration number; date of incidence; topography; grading; staging	Uses ICD codes for cancer recording
Dow Cancer registry	Data from biopsies recorded digitally.	In-house built software	Duplicate and irrelevant entries sorted during data cleaning.	site; sex; age	Uses ICD-10 for cancer coding
Tumor registry at Allied Hospital, Faisalabad	–	–	–	age; tumor type; site association.	–
Central Nervous System Tumour registry at Combined Military Hospital, Rawalpindi	–	–	–	–	–
Nishtar Medical University Hospital Multan (NMH) Registry	–	–	–	–	–
Shifa International Hospital, Islamabad (SIH) registry	Patient medical records and hospital databases.	–	–	–	–
Punjab Institute of Nuclear Medicine (PINUM) Cancer Registry	Data from medical records, recorded in registry files	–	–	number; sex; age; date of incidence; history with diabetes and hypertension; family history; site address; cancer type; subtype	–
Liaquat National Hospital and Medical College Hematologic Tumour Registry	Data obtained from medical records, that are filled by resident physicians on predesigned questionnaire.	–	–	patients MR numbers; name; age; sex; address; contact numbers; ethnicity; family history for haematological malignancies; tumor name; tumor stage	–
Patel hospital Cancer Registry Database	Data was collected using a routine registry form.	–	–	stage; tumour type; size; grade margin; depth of invasion; perineural invasion; bone involvement; vessel invasion; muscle invasion; extracapsular spread; gender; age; comorbidities; addition	–

a Data variables outline the ones that were extracted from the published studies reporting data from these registries. This list is not comprehensive and does not include all the variables recorded in the registry.

### Source of funding, activity status, research output, strengths, and limitations of cancer registries in Pakistan

**Fig. 2:**
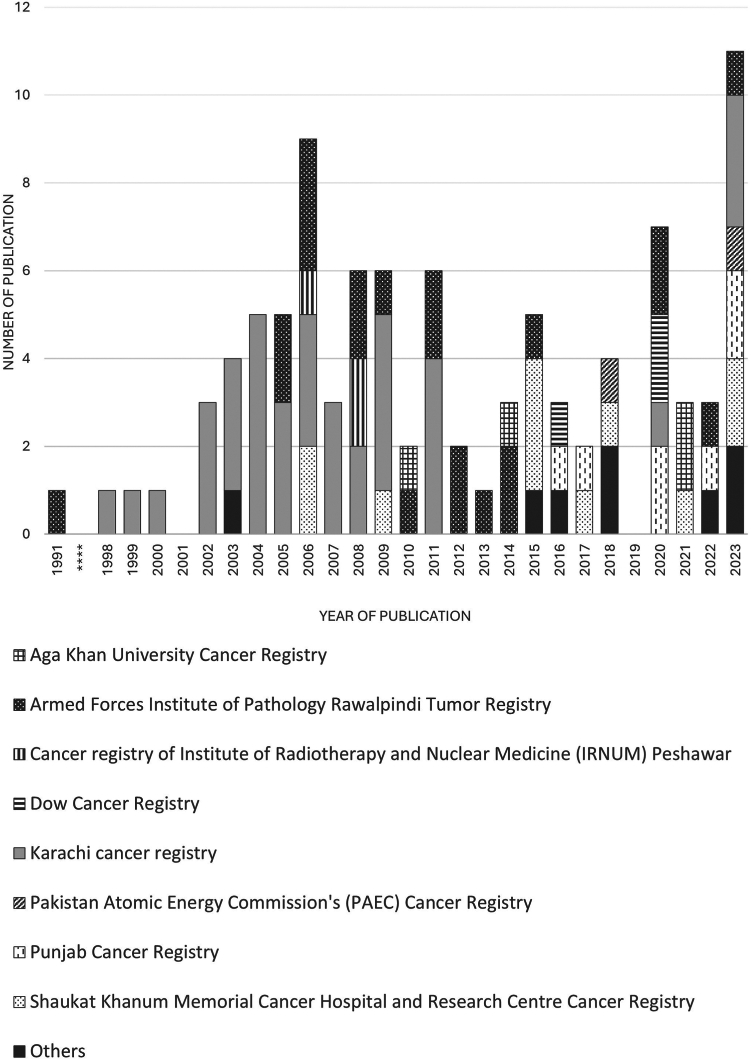
Number of publications by cancer registries in Pakistan over the years.

Information on the sources of funding for cancer registries in Pakistan was inconsistent. The KCR is supported by government funds and international collaborations, notably with the International Agency for Research on Cancer (IARC). The PCR is sponsored by the Shaukat Khanum Memorial Trust. Other registries did not specify their funding sources. Of the 17 registries identified, 7 are currently active. There is notable variation in the research output of these registries, with most publications (n = 37, 36.3%) from KCR and 21 (21.6%) from Armed Forces Institute of Pathology Tumor Registry (AFIPR-TR) (Table 3). A report consolidating data from multiple regional and institutional registries presents it as a national cancer registry, although this report does not provide a fully representative dataset for the entire country. The number of articles reporting data from the registries over the years is shown in Fig. 2. The strengths and limitations of each registry are outlined in Table 4.

**Table 3:** Source of funding, current status, and research output.

Registry	Source of funding	Registry active (Yes/No)	Number of articles reporting data from the registry
Karachi Cancer Registry	Funding is primarily through government support and collaboration with international agencies such as the IARC.	Yes	37
Armed Forces Institute of Pathology Rawalpindi Tumor Registry	Not specified	Yes	22
Shaukat Khanum Memorial Cancer Hospital and Research Centre (SKMCH & RC)'s Hospital Cancer Registry	Not specified	Yes	11
Punjab Cancer Registry	Sponsored by the Shaukat Khanum Memorial Trust	Yes	7
Aga Khan University	Not specified	Yes	4
Dow Cancer registry	Not specified	Yes	3
Cancer Registry of Institute of Radiotherapy and Nuclear Medicine (IRNUM) Peshawar	Not specified	Not specified	3
Pakistan Atomic Energy Commission's (PAEC) Cancer Registry	Not specified	Not specified	2
Tumor Registry at Jinnah Hospital	Not specified	Not specified	1
Tumor registry at Allied Hospital, Faisalabad	Not specified	Yes	1
Central Nervous System Tumour registry at Combined Military Hospital, Rawalpindi	Not specified	Not specified	1
Nishtar Medical University Hospital Multan (NMH) registry	Not specified	Not specified	1
Shifa International Hospital, Islamabad (SIH) registry	Not specified	Not specified	1
Punjab Institute of Nuclear Medicine (PINUM) Cancer Registry	Not specified	Not specified	1
Liaquat National Hospital and Medical College Hematologic Tumour registry	Not specified	Not specified	1
Patel hospital cancer registry database	Not specified	Not specified	1
National Cancer Registry	Not specified	No	0

Note: The numbers of articles in the last column of this table represent the number of studies reporting data from each registry. Some studies reported data from multiple registries and were counted once per registry, which may result in a total count higher than the actual number of studies. Additionally, studies that described registry characteristics but did not report data from the registry have been excluded from this table.

**Table 4:** Strengths and limitations.

Registry	Strengths	Limitations
Karachi Cancer Registry	• Records the epidemiology and burden of cancer cases in Karachi to estimate the national cancer burden. • Comprehensive data collection from multiple sources, with improved accuracy and completion. • Use of CANREG-3 software enhances data validity. • Population-based descriptive epidemiology data is provided. • Voting member of the International Association of Cancer Registries (IACR)	• Under-registration of clinically diagnosed cases due to incomplete records and inadequate death registration procedures. • Possibility of missing cases not entering the healthcare system. • Reporting of new cancer cases is not mandatory, leading to potential data gaps and duplication. • Tumor coding errors and lack of computer training in early years. • High rates of duplication, especially in the early years of the registry. • Patients are frequently lost to follow-up. • Reliable data acquisition is challenging due to variations in practices at different hospitals.
Armed Forces Institute of Pathology Rawalpindi Tumor Registry	• Data collection on biopsy-proven tumor cases • Standardized reporting using ICD-O codes	• Mainly provides relative frequency data, which limits statistical analysis • Lack of patient follow-up
Shaukat Khanum Memorial Cancer Hospital and Research Centre (SKMCH & RC)'s Hospital Cancer Registry	• Real-time data collection • Data covering clinical, research, and administrative sections	• Inconsistent staging usage • Lack of risk factor data recording
Punjab Cancer Registry	• Data collection from multiple collaborating centers. • Use of electronic and mobile data applications for efficient data management. • Member of the International Association of Cancer Registries (awarded Associate Member status in November 2011). • Utilizes the International Classification of Diseases for recording cancer data.	• Loss of contact with patients on follow-up. • Incomplete data reporting, with not all numbers reported. • Lack of data linkages with the National Database and Registration Authority, leading to challenges in estimating mortality/survival rates.
Aga Khan University Pathology-based Cancer Registry	• Extensive geographical coverage with data from 84 centers. • Regular updates and validity checks. • Association with international cancer registries (IACR). • Provides comprehensive cancer surveillance across Pakistan and contribute to national and international cancer registries.	• Few articles reporting data from the registry, it may indicate limited utilization or visibility of the registry data in the scientific community, despite good collection and validity checks. • Challenging consistent follow-up with patients affecting the accuracy of long-term data.
Dow Cancer registry	• Based at the largest public sector hospital in Karachi, serving as the major public-sector stakeholder in cancer diagnostics in the city. • City-wide collection centers ensure samples are representative of all districts of Karachi. • Karachi's multi-ethnic population, often called “mini-Pakistan,” potentially reflects country-wide cancer statistics.	• Missing cancer patients at other oncology/diagnostic centers of Karachi.
Cancer Registry of Institute of Radiotherapy and Nuclear Medicine (IRNUM) Peshawar	• Data collected by qualified and experienced technical staff • Data utilized to examine current cancer trends and forecast future trends	• No follow-up, hence, no data available on mortality or survival rates
Pakistan Atomic Energy Commission's (PAEC) Cancer Registry	• Comprehensive national coverage with data collected from multiple centers across the country. • Collaboration with specialized nuclear medicine and oncology divisions • Aims at utilizing data for devising cancer control and prevention strategies	• May not capture all incident cancers within Pakistan. • Difficult to assess mortality rates and not true representative of the country population due to lack of data-entries from pathology labs, discharge abstracts of hospitals and death certificates. • No means of detecting duplicate cases. • Data included ill-defined and lost to follow up cases.
Tumor Registry at Jinnah Hospital	• Patient-level data • Data updated on subsequent patient visits	• Limited to patients visiting Jinnah Hospital, thus not representative of the entire population
Tumor registry at Allied Hospital, Faisalabad	• Data on cancer patients within a specific hospital setting.	• Despite multiple studies reporting data from the registry there is limited information available about establishment, data collection methods, functioning of the registry.
Central Nervous System Tumour registry at Combined Military Hospital, Rawalpindi	• Data on cancer patients within a specific hospital setting.	• Lost to follow-up cases • Incomplete medical records • Limited information available about establishment, data collection methods, and functioning of the registry
Nishtar Medical University Hospital Multan (NMH) registry	• Data on cancer patients within a specific hospital setting.	• Lack of detailed information on data variables, data collection methods, and software used. • Limited population coverage beyond the hospital setting.
Shifa International Hospital, Islamabad (SIH) registry	• Data on cancer patients within a specific hospital setting.	• Lack of detailed information on the population size and exact data variables. • Limited to data from a single hospital, which may not be representative of the broader population.
Punjab Institute of Nuclear Medicine (PINUM) Cancer Registry	• Contributes to a larger cancer registry in the country	• Specific details on registry activity and software are not available
Liaquat National Hospital and Medical College Hematologic Tumour registry	• Data on cancer patients within a specific hospital setting. • Contributes to country haematological data which is reported rarely.	• Limited number of patients included in registry • Lack of data to ascertain etiological factors • Absence of follow-up data
Patel hospital cancer registry database	• Data on cancer patients within a specific hospital setting.	• Lack of follow-up information
National Cancer Registry	• Not specified	• The registry could not sustain due to funding issues.

## Discussion

The global cancer burden is rising, posing significant challenges for south and southeast Asian countries.^17^ Accurate cancer data is crucial for effective control and policymaking. However, the absence of a unified national cancer registry in Pakistan limits the collection of comprehensive nationwide data.^18^ Despite this limitation, Pakistan has established a network of 17 cancer registries employing varied data collection methods, including paper-based forms, mobile applications, and software like Oracle-based Hospital Information Systems and CANREG-3. Although funding is limited and research output varies, these registries contribute valuable data for cancer surveillance.

Despite existing challenges, Pakistan's cancer registries collect high-quality data from different regions, providing a valuable foundation for studying cancer trends and outcomes across the country. Key registries, such as SKMCH&RC, PCR, and KCR, ensure data quality through duplication prevention and validation measures. These registries record essential information, including patient demographics, tumour characteristics, and outcomes, enabling in-depth analysis of cancer patterns. Integrating registry data could improve geographic coverage, offering a nationally representative overview of cancer and facilitating comparisons between regions and subpopulations.

**Fig. 3:**
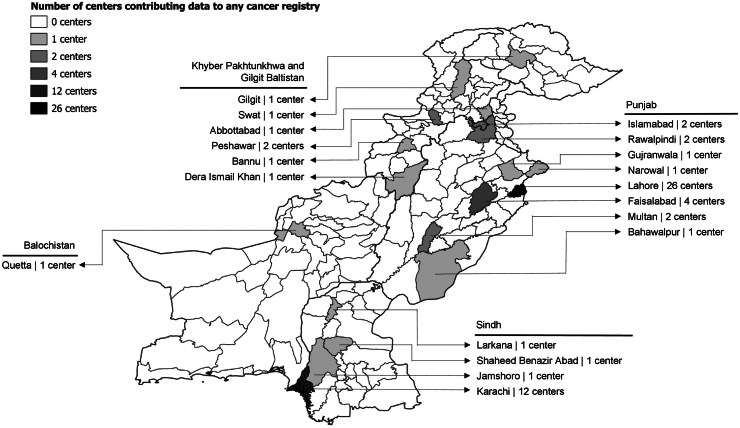
Number of centers contributing to at least one cancer registry in cities across Pakistan.

Only 19 out of 129 cities contribute data to cancer registries (Fig. 3), highlighting significant geographic underrepresentation. Among the 62 cancer centers submitting data, 52 are in urban areas, 10 in smaller towns, and none in rural institutions, further emphasising the gap in rural cancer data. While patients from rural areas may travel to urban centers for care and be included in registries, most rural populations in Pakistan often lack the resources to do so. As a result, many cases may remain undocumented. Other limitations explicitly acknowledged by authors of included studies include data incompleteness, lack of representativeness, selection bias, and deviations from international reporting standards.

Incomplete hospital records and voluntary participation in registries, such as KCR and SKMCH&RC, contribute to under-registration. Clinically diagnosed cases often go unrecorded. This issue is compounded by resource constraints and low institutional engagement. Additionally, the absence of data linkage between cancer registries and the National Database and Registration Authority (NADRA) hinders accurate mortality and survival rate estimation^19^ and increases the risk of data duplication.^20^ Furthermore, patient mobility complicates registry population determination, as individuals often seek treatment at multiple hospitals. Limited funding and reliance on individual efforts make registries vulnerable, demonstrated by KCR's five-year hiatus.^20^ The shortage of trained personnel and lack of national coordination across registries further restrict effective cancer surveillance.^21^

Establishing and maintaining a national cancer registry requires significant financial investment in infrastructure, technology, personnel, and operations. In LMICs like Pakistan, budget constraints pose a major challenge. Costs depend on factors such as population size, data collection methods (paper vs. electronic), case volume, data quality, reporting sources, staff turnover, and local cost of living.

Cancer registry costs in LMICs are generally low, averaging less than 1 cent per person in India, 1 cent in Uganda, and 3 cents in Kenya.^22^ In Pakistan, assessing current systems to identify cost drivers and collaborating with international agencies could help manage expenses. Studies in other LMICs estimate costs per registered cancer case ranging from US$3.77 in Mumbai to US$15.62 in Nairobi.^23^ Assuming costs similar to those in India, at approximately US$3.77 per case, and considering an estimated 185,748 new cancer cases annually in Pakistan, a national cancer registry would require an annual budget of just over US$700,000 (Note: US$ 3.77 = around 321 INR and 1065 PKR; US$15.62 = around 2015 KES; US$700,000 = around 197,904,000 PKR [as of May 25, 2025]). The economic benefits of adequately resourced cancer control efforts would far exceed this investment.^24^

Global examples of national cancer registries provide valuable lessons for Pakistan. The Surveillance, Epidemiology, and End Results (SEER) Program in the United States,^25^^,^^26^ demonstrates the benefits of data compatibility, national pooling, and technological tools such as web-based State Cancer Profiles^27^ for data analysis, accessibility and public health planning.^28^ The UK's National Cancer Registration and Analysis Service (NCRAS) integrates data from multiple sources, linking cancer registry data with hospital records and patient surveys.^29^ Similarly, Australia's Australian Cancer Database^30^ exemplifies regional coordination and standardisation.

In south Asia, India's National Cancer Registry Program (NCRP), includes 26 population-based and 7 hospital-based registries.^31^ However, while it covers urban populations, it lacks full national representation and data consistency. Sri Lanka's National Cancer Control Programme has been monitoring cancer trends since 1985 through a hospital-based registry, publishing data up to 2005.^32^ Learning from these models, Pakistan can adopt a similar approach to improve its cancer surveillance efforts by adopting standardised data collection, leveraging technological innovations for better data management, and utilising registry data to guide public health strategies.

A structured implementation roadmap is essential for a standardised, non-duplicative national cancer registry. Establishing a National Cancer Registry authority under the Ministry of Health would provide centralised governance, uniform data collection, and system-wide integration. This authority should mandate cancer reporting from all oncology institutions. Sustained financial investment, the adoption of innovative technologies, training programs, and expertise from existing registries can foster collaboration and address local challenges. Linking the registry with NADRA using unique patient identifiers would prevent data duplication, improve patient tracking and facilitate survival outcomes analysis. Standardised data-sharing protocols should be enforced across hospitals, regional cancer centers, and pathology labs to ensure data accuracy and consistency.

A phased implementation approach is recommended. Initially, a pilot program should be launched in major oncology centers to establish reporting mechanisms, train personnel, and test data linkages across centers. Coverage should then be expanded to regional hospitals and underserved areas, using mHealth and Telemedicine platforms to improve access. Nationwide integration should be supported through public-private partnerships and sustainable funding models for long-term viability. Adopting best practices and collaborating with global networks like IARC^33^ would enhance data quality and global comparability. Regular audits, external validation, and collaboration with international registries will further strengthen data reliability and registry credibility.

This study relied on published literature, registry websites, and reports, where critical information, such as data collection methods, population coverage, and operational details, was often unavailable. Some registries collect data but do not publish it, meaning they could have been overlooked. Additionally, the lack of auditing reports limits insights into data quality and accuracy.

Furthermore, much of the research on cancer registries in Pakistan has been published in less widely recognised peer-reviewed journals, often in local publications. While these studies provide valuable insights, the limited availability of internationally recognised references should be considered when interpreting findings. Nonetheless, we chose to include these articles in our Review, as they offer crucial, and often the only, insight into the state of cancer registries in Pakistan.

## Conclusion

This Review represents a critical step in understanding the state of cancer registries in Pakistan, revealing a fragmented landscape with significant gaps in data collection, quality, and coverage. A national, unified cancer registry in Pakistan could not only transform cancer data collection but also inform public policy and improve cancer control. By prioritising collaboration and sustainable investment, Pakistan can strengthen regional cancer surveillance, contributing to evidence-based cancer control strategies applicable across the region.

**Search strategy and selection criteria**

The review team collaborated with a research librarian (L.C.) to develop a comprehensive search strategy using controlled vocabulary and title or abstract terms related to cancer registries in Pakistan. Full Boolean search strings are provided in Supplementary Table S2. Searches were conducted on June 21, 2023, from database inception to incorporate the latest available literature, across multiple databases, including MEDLINE (EBSCO), PubMed, Cochrane Central Register of Controlled Trials (Wiley), Scopus (Embase records only), PakMediNet, Web of Science Core Collection (Clarivate), and Google Scholar (top 200 relevancy-sorted results). These databases were chosen for their broad coverage of biomedical and regional literature. Search results were downloaded into EndNote, manually deduplicated using the Bramer method,^34^ and uploaded to Covidence for screening. Supplementary searches were conducted using identified cancer registry names from the initial screening. Related websites, where available, were reviewed for additional reports or data not captured in traditional literature, providing further insights.

We included studies that reported primary data from any cancer registry in Pakistan, irrespective of age, gender, cancer type, or stage. A cancer registry was defined as an organised system using observational methods to collect uniform cancer data for scientific, clinical, or policy purposes. We considered all reported regional, city, and institution-level registries. Quantitative studies presenting cancer patient data, reviews, texts, and short communications detailing the structure and functioning of cancer registries in Pakistan, were included. No language restrictions were applied. We excluded articles if they reported data from hospital records without representing a formal registry, used data from non-cancer registries, were general discussions on cancer registration deficiencies without specific data, or consisted solely of abstracts.

## Contributors

SSV: Data Collection, Data curation, Methodology, Project administration, Writing—original draft, Writing—review & editing.

KSA: Methodology, Project administration, Writing—review & editing.

MS and MH: Data Collection, Methodology, Writing—review & editing.

LC: Literature Search, Methodology, Writing—review & editing.

FA, SP, ZF, and AB: Writing—review & editing.

SNZ: Conceptualisation, Supervision, Writing—review & editing.

## Data sharing statement

The data supporting the findings of this study are derived from publicly accessible sources, including published literature, registry websites, and online reports related to cancer registries in Pakistan. Primary data from individual registries are not publicly available. Specific data requests can be made to the corresponding author.

## Editor note

The Lancet Group takes a neutral position with respect to territorial claims in published maps and institutional affiliations.

## Declaration of interests

SNZ receives partial salary support for research from the NIH/NCI Early-Stage Surgeon Scientist Program Grant P30 CA014520-48S4. We declare no other competing interests.
