# Supplementary material for: Cancer registries in Pakistan: a scoping review
Source: Lancet Reg Health Southeast Asia. 2025 Jun 14;38:100615. doi: 10.1016/j.lansea.2025.100615 (PMC12205766; doi:10.1016/j.lansea.2025.100615)
Supplement: Supplementary Tables [file mmc1.docx]

**Supplementary Tables**

1. Supplementary Table 1: Preferred Reporting Items for Systematic reviews and Meta-Analyses extension for Scoping Reviews (PRISMA-ScR) Checklist.
2. Supplementary Table 2: Search strategy for each database
3. Supplementary Table 3: List of extracted variables from each study.
4. Supplementary Table 4: Summary of studies included in the scoping review.
5. Supplementary Table 5: Collaborating centers for multi-institutional level registries
6. References for the included studies

**Supplementary Table 1: Preferred Reporting Items for Systematic reviews and Meta-Analyses extension for Scoping Reviews (PRISMA-ScR) Checklist.**

| **SECTION** | **ITEM** | **PRISMA-ScR CHECKLIST ITEM** | **REPORTED ON PAGE #** |
| --- | --- | --- | --- |
| **TITLE** | | | |
| Title | 1 | Identify the report as a scoping review. | Page 1 |
| **ABSTRACT** | | | |
| Structured summary | 2 | Provide a structured summary that includes (as applicable): background, objectives, eligibility criteria, sources of evidence, charting methods, results, and conclusions that relate to the review questions and objectives. | Page 2 |
| **INTRODUCTION** | | | |
| Rationale | 3 | Describe the rationale for the review in the context of what is already known. Explain why the review questions/objectives lend themselves to a scoping review approach. | Page 3 |
| Objectives | 4 | Provide an explicit statement of the questions and objectives being addressed with reference to their key elements (e.g., population or participants, concepts, and context) or other relevant key elements used to conceptualize the review questions and/or objectives. | Page 3 |
| **METHODS** | | | |
| Protocol and registration | 5 | Indicate whether a review protocol exists; state if and where it can be accessed (e.g., a Web address); and if available, provide registration information, including the registration number. | Page 3 |
| Eligibility criteria | 6 | Specify characteristics of the sources of evidence used as eligibility criteria (e.g., years considered, language, and publication status), and provide a rationale. | Page 4 |
| Information sources* | 7 | Describe all information sources in the search (e.g., databases with dates of coverage and contact with authors to identify additional sources), as well as the date the most recent search was executed. | Page 4 |
| Search | 8 | Present the full electronic search strategy for at least 1 database, including any limits used, such that it could be repeated. | Page 4 and Supplement table 2 |
| Selection of sources of evidence† | 9 | State the process for selecting sources of evidence (i.e., screening and eligibility) included in the scoping review. | Page 4 |
| Data charting process‡ | 10 | Describe the methods of charting data from the included sources of evidence (e.g., calibrated forms or forms that have been tested by the team before their use, and whether data charting was done independently or in duplicate) and any processes for obtaining and confirming data from investigators. | Page 4 |
| Data items | 11 | List and define all variables for which data were sought and any assumptions and simplifications made. | Page 4 |
| Critical appraisal of individual sources of evidence§ | 12 | If done, provide a rationale for conducting a critical appraisal of included sources of evidence; describe the methods used and how this information was used in any data synthesis (if appropriate). | Page 4 |
| Synthesis of results | 13 | Describe the methods of handling and summarizing the data that were charted. | Page 4-5 |
| **RESULTS** | | | |
| Selection of sources of evidence | 14 | Give numbers of sources of evidence screened, assessed for eligibility, and included in the review, with reasons for exclusions at each stage, ideally using a flow diagram. | Page 5 |
| Characteristics of sources of evidence | 15 | For each source of evidence, present characteristics for which data were charted and provide the citations. | Page 6 |
| Critical appraisal within sources of evidence | 16 | If done, present data on critical appraisal of included sources of evidence (see item 12). | Page 6 |
| Results of individual sources of evidence | 17 | For each included source of evidence, present the relevant data that were charted that relate to the review questions and objectives. | Supplement table 2 |
| Synthesis of results | 18 | Summarize and/or present the charting results as they relate to the review questions and objectives. | Page 5-9 |
| **DISCUSSION** | | | |
| Summary of evidence | 19 | Summarize the main results (including an overview of concepts, themes, and types of evidence available), link to the review questions and objectives, and consider the relevance to key groups. | Page 10-12 |
| Limitations | 20 | Discuss the limitations of the scoping review process. | Page 12 |
| Conclusions | 21 | Provide a general interpretation of the results with respect to the review questions and objectives, as well as potential implications and/or next steps. | Page 12 |
| **FUNDING** | | | |
| Funding | 22 | Describe sources of funding for the included sources of evidence, as well as sources of funding for the scoping review. Describe the role of the funders of the scoping review. | None |

**Supplementary Table 2: Search strategy for each database**

| **DATABASE** | **EBSCO Medline** | | |
| --- | --- | --- | --- |
| **Date** | 06/21/2023 | | |
| **Results** | 1910 | | |
| **Strategy** | #1 AND #2 AND #3 | | |
| #1 | (MH "Neoplasms+" OR TI ( neoplas* OR cancer* OR oncolog* OR malignan* OR metasta* OR carcinom* OR adenocarcinom* OR adenom* OR leukemia* OR leukaemia* OR lymphoma* OR myeloma* OR tumor* OR tumour* OR sarcoma* OR osteosarcoma*) OR AB (neoplas* OR cancer* OR oncolog* OR malignan* OR metasta* OR carcinom* OR adenocarcinom* OR adenom* OR leukemia* OR leukaemia* OR lymphoma* OR tumor* OR tumour* OR sarcoma* OR osteosarcoma*)) | | |
| #2 | (MH “Registries” OR TI (registr*) OR AB (registr*) OR TI (( neoplas* OR cancer* OR oncolog* OR malignan* OR metasta* OR carcinom* OR adenocarcinom* OR adenoma* OR leukemia* OR leukaemia* OR lymphoma* OR myeloma* OR tumor* OR tumour* OR sarcoma* OR osteosarcoma* OR national OR region* OR provinc*) N7 (incidence OR prevalence OR burden OR epidemiol* OR data* OR report*)) OR AB ((neoplas* OR cancer* OR oncolog* OR malignan* OR metasta* OR carcinom* OR adenocarcinom* OR adenoma* OR leukemia* OR leukaemia* OR lymphoma* OR myeloma* OR tumor* OR tumour* OR sarcoma* OR osteosarcoma* OR national OR region* OR provinc*) N7 (incidence OR prevalence OR burden OR epidemiol* OR data* OR report*)) OR TI ((institution* OR hospital OR hospitals OR facilit*) N7 (data* OR report*)) OR AB ((institution* OR hospital OR hospitals OR facilit*) N7 (data* OR report*))) | | |
| #3 | (MH (“Pakistan+”) OR TI (Pakistan*) OR AB (Pakistan*) OR AF (Pakistan*)) | | |
| **DATABASE** | **PubMed: Non-Medline citations** | | |
| **Date** | 06/21/2023 | | |
| **Results** | 352 | | |
| **Strategy** | (#1 AND #2 AND #3) NOT #4 | | |
| #1 | ("Neoplasms"[mesh] OR neoplas*[tiab] OR cancer*[tiab] OR oncolog*[tiab] OR malignan*[tiab] OR metasta*[tiab] OR carcinom*[tiab] OR adenocarcinom*[tiab] OR adenoma*[tiab] OR leukemia*[tiab] OR leukaemia*[tiab] OR lymphoma*[tiab] OR myeloma*[tiab] OR tumor*[tiab] OR tumour*[tiab] OR sarcoma*[tiab] OR osteosarcoma*[tiab]) | | |
| #2 | ("Registries"[mesh:noexp] OR registr*[tiab] OR "neoplasm incidence"[tiab:~7] OR "neoplasm prevalence"[tiab:~7] OR "neoplasm burden"[tiab:~7] OR "neoplasm epidemiology"[tiab:~7] OR "neoplasms incidence"[tiab:~7] OR "neoplasms prevalence"[tiab:~7] OR "neoplasms burden"[tiab:~7] OR "neoplasms epidemiology"[tiab:~7] OR "cancer incidence"[tiab:~7] OR "cancer prevalence"[tiab:~7] OR "cancer burden"[tiab:~7] OR "cancer epidemiology"[tiab:~7] OR "cancers incidence"[tiab:~7] OR "cancers prevalence"[tiab:~7] OR "cancers burden"[tiab:~7] OR "cancers epidemiology"[tiab:~7] OR "oncological incidence"[tiab:~7] OR "oncological prevalence"[tiab:~7] OR "oncological burden"[tiab:~7] OR "oncological epidemiology"[tiab:~7] OR "oncology incidence"[tiab:~7] OR "oncology prevalence"[tiab:~7] OR "oncology burden"[tiab:~7] OR "oncology epidemiology"[tiab:~7] OR “malignancy incidence”[tiab:~7] OR “malignancy prevalence"[tiab:~7] OR "malignancy burden"[tiab:~7] OR "malignancy epidemiology"[tiab:~7] OR "malignancies incidence"[tiab:~7] OR " malignancies prevalence"[tiab:~7] OR " malignancies burden"[tiab:~7] OR "malignancies epidemiology"[tiab:~7] OR "metastases incidence"[tiab:~7] OR "metastases prevalence"[tiab:~7] OR "metastases burden"[tiab:~7] OR "metastases epidemiology"[tiab:~7] OR "metastasis incidence"[tiab:~7] OR "metastasis prevalence"[tiab:~7] OR "metastasis burden"[tiab:~7] OR "metastasis epidemiology"[tiab:~7] OR "adenocarcinoma incidence"[tiab:~7] OR "adenocarcinoma prevalence"[tiab:~7] OR "adenocarcinoma burden"[tiab:~7] OR "adenocarcinoma epidemiology"[tiab:~7] OR "adenocarcinomas incidence"[tiab:~7] OR "adenocarcinomas prevalence"[tiab:~7] OR "adenocarcinomas burden"[tiab:~7] OR "adenocarcinomas epidemiology"[tiab:~7] OR "carcinoma incidence"[tiab:~7] OR "carcinoma prevalence"[tiab:~7] OR "carcinoma burden"[tiab:~7] OR "carcinoma epidemiology"[tiab:~7] OR "carcinomas incidence"[tiab:~7] OR "carcinomas prevalence"[tiab:~7] OR "carcinomas burden"[tiab:~7] OR "carcinomas epidemiology"[tiab:~7] OR "adenoma incidence"[tiab:~7] OR "adenoma prevalence"[tiab:~7] OR "adenoma burden"[tiab:~7] OR "adenoma epidemiology"[tiab:~7] OR "adenomas incidence"[tiab:~7] OR "adenomas prevalence"[tiab:~7] OR "adenomas burden"[tiab:~7] OR "adenomas epidemiology"[tiab:~7] OR "leukemia incidence"[tiab:~7] OR "leukemia prevalence"[tiab:~7] OR "leukemia burden"[tiab:~7] OR "leukemia epidemiology"[tiab:~7] OR "leukemias incidence"[tiab:~7] OR "leukemias prevalence"[tiab:~7] OR "leukemias burden"[tiab:~7] OR "leukemias epidemiology"[tiab:~7] OR "leukaemia incidence"[tiab:~7] OR "leukaemia prevalence"[tiab:~7] OR "leukaemia burden"[tiab:~7] OR "leukaemia epidemiology"[tiab:~7] OR "leukaemias incidence"[tiab:~7] OR "leukaemias prevalence"[tiab:~7] OR "leukaemias burden"[tiab:~7] OR "leukaemias epidemiology"[tiab:~7]OR "lymphoma incidence"[tiab:~7] OR "lymphoma prevalence"[tiab:~7] OR "lymphoma burden"[tiab:~7] OR "lymphoma epidemiology"[tiab:~7] OR "lymphomas incidence"[tiab:~7] OR "lymphomas prevalence"[tiab:~7] OR "lymphomas burden"[tiab:~7] OR "lymphomas epidemiology"[tiab:~7] OR "myeloma incidence"[tiab:~7] OR "myeloma prevalence"[tiab:~7] OR "myeloma burden"[tiab:~7] OR "myeloma epidemiology"[tiab:~7] OR "myelomas incidence"[tiab:~7] OR "myelomas prevalence"[tiab:~7] OR "myelomas burden"[tiab:~7] OR "myelomas epidemiology"[tiab:~7] OR "tumor incidence"[tiab:~7] OR "tumor prevalence"[tiab:~7] OR "tumor burden"[tiab:~7] OR "tumor epidemiology"[tiab:~7] OR "tumors incidence"[tiab:~7] OR "tumors prevalence"[tiab:~7] OR "tumors burden"[tiab:~7] OR "tumors epidemiology"[tiab:~7] OR "tumour incidence"[tiab:~7] OR "tumour prevalence"[tiab:~7] OR "tumour burden"[tiab:~7] OR "tumour epidemiology"[tiab:~7] OR "tumours incidence"[tiab:~7] OR "tumours prevalence"[tiab:~7] OR "tumours burden"[tiab:~7] OR "tumours epidemiology"[tiab:~7] OR "sarcoma incidence"[tiab:~7] OR "sarcoma prevalence"[tiab:~7] OR "sarcoma burden"[tiab:~7] OR "sarcoma epidemiology"[tiab:~7] OR "sarcomas incidence"[tiab:~7] OR "sarcomas prevalence"[tiab:~7] OR "sarcomas burden"[tiab:~7] OR "sarcomas epidemiology"[tiab:~7] OR "osteosarcoma incidence"[tiab:~7] OR "osteosarcoma prevalence"[tiab:~7] OR "osteosarcoma burden"[tiab:~7] OR "osteosarcoma epidemiology"[tiab:~7] OR "osteosarcomas incidence"[tiab:~7] OR "osteosarcomas prevalence"[tiab:~7] OR "osteosarcomas burden"[tiab:~7] OR "osteosarcomas epidemiology"[tiab:~7] OR "neoplasm data"[tiab:~7] OR "neoplasm database"[tiab:~7] OR “neoplasm reporting”[tiab:~7] OR "neoplasms data"[tiab:~7] OR "neoplasms database"[tiab:~7] OR “neoplasms reporting”[tiab:~7] OR "cancer data"[tiab:~7] OR "cancer database"[tiab:~7] OR "cancer reporting"[tiab:~7] OR "cancers data"[tiab:~7] OR "cancers database"[tiab:~7] OR "cancers reporting"[tiab:~7] OR "oncology data"[tiab:~7] OR "oncology database"[tiab:~7] OR “oncology reporting”[tiab:~7] OR "oncological data"[tiab:~7] OR "oncological database"[tiab:~7] OR “oncological reporting”[tiab:~7] OR "tumor data"[tiab:~7] OR "tumor database"[tiab:~7] OR “tumor reporting”[tiab:~7] OR "tumors data"[tiab:~7] OR "tumors database"[tiab:~7] OR “tumors reporting”[tiab:~7] OR "tumour data"[tiab:~7] OR "tumour database"[tiab:~7] OR “tumour reporting”[tiab:~7] OR "tumours data"[tiab:~7] OR "tumours database"[tiab:~7] OR “tumours reporting”[tiab:~7] OR "national data"[tiab:~7] OR "national database"[tiab:~7] OR “national reporting”[tiab:~7] OR "region data"[tiab:~7] OR "region database"[tiab:~7] OR “region reporting”[tiab:~7] OR "regional data"[tiab:~7] OR "regional database"[tiab:~7] OR “regional reporting”[tiab:~7] OR "province data"[tiab:~7] OR "province database"[tiab:~7] OR “province reporting”[tiab:~7] OR "provincial data"[tiab:~7] OR "provincial database"[tiab:~7] OR “provincial reporting”[tiab:~7] OR "institution data"[tiab:~7] OR "institution database"[tiab:~7] OR “institution reporting”[tiab:~7] OR "institutional data"[tiab:~7] OR "institutional database"[tiab:~7] OR “institutional reporting”[tiab:~7] OR "hospital data"[tiab:~7] OR "hospital database"[tiab:~7] OR “hospital reporting”[tiab:~7] OR "hospitals data"[tiab:~7] OR "hospitals database"[tiab:~7] OR “hospitals reporting”[tiab:~7] OR "facility data"[tiab:~7] OR "facility database"[tiab:~7] OR “facility reporting”[tiab:~7] OR "facillities data"[tiab:~7] OR "facilities database"[tiab:~7] OR “facilities reporting”[tiab:~7]) | | |
| #3 | ("Pakistan"[mesh] OR Pakistan*[tiab] OR Pakistan*[affil]) | | |
| #4 | medline[filter] | | |
| **DATABASE** | **Scopus (Embase only)** | | |
| **Date** | 06/21/2023 | | |
| **Results** | 2079 | | |
| **Strategy** | #1 AND #2 AND #3 AND #4 | | |
| #1 | TITLE-ABS-KEY(neoplas* OR cancer* OR oncolog* OR malignan* OR metasta* OR carcinom* OR adenocarcinom* OR adenom* OR leukemia* OR leukaemia* OR lymphoma* OR myeloma* OR tumor* OR tumour* OR sarcoma* OR osteosarcoma*) | | |
| #2 | TITLE-ABS-KEY(registr* OR ((neoplas* OR cancer* OR malignan* OR oncolog* OR carcinom* OR adenocarcinom* OR adenoma* OR leukemia* OR leukaemia* OR lymphoma* OR myeloma* OR tumor* OR tumour* OR sarcoma* OR osteosarcoma* OR national OR region* OR provinc*) W/7 (incidence OR prevalence OR burden OR epidemiol* OR data* OR report*)) OR ((institution* OR hospital OR hospitals OR facilit*) W/7 (data* OR report*))) | | |
| #3 | (TITLE-ABS-KEY(Pakistan*) OR AFFIL(pakistan*)) | | |
| #4 | INDEX(embase) | | |
| **DATABASE** | **Web of Science Core Collection: Science Citation Index-Expanded, Emerging Sources Citation Index** | | |
| **Date** | 06/21/2023 | | |
| **Results** | 2467 | | |
| **Strategy** | #1 AND #2 AND #3 | | |
| #1 | TS=(neoplas* OR cancer* OR oncolog* OR malignan* OR metasta* OR carcinom* OR adenocarcinom* OR adenom* OR leukemia* OR leukaemia* OR lymphoma* OR myeloma* OR tumor* OR tumour* OR sarcoma* OR osteosarcoma*) | | |
| #2 | TS=(registr* OR ((neoplas* OR cancer* OR oncolog* OR malignan* OR metasta* OR carcinom* OR adenocarcinom* OR adenoma* OR leukemia* OR leukaemia* OR lymphoma* OR myeloma* OR tumor* OR tumour* OR sarcoma* OR osteosarcoma*) NEAR/7 (incidence OR prevalence OR burden OR epidemiol*)) OR ((neoplas* OR cancer* OR oncolog* OR tumor* OR tumour* OR oncolog* OR national* OR region* OR provinc* OR institution* OR hospital OR hospitals OR facilit*) NEAR/7 (data OR database* OR report*))) | | |
| #3 | (TS=(Pakistan*) OR CU=(pakistan*)) | | |
| **DATABASE** | **Web of Science Preprint Citation Index** | | |
| **Date** | 06/21/2023 | | |
| **Results** | 10 | | |
| **Strategy** | #1 AND #2 AND #3 | | |
| #1 | TS=(neoplas* OR cancer* OR oncolog* OR malignan* OR metasta* OR carcinom* OR adenocarcinom* OR adenom* OR leukemia* OR leukaemia* OR lymphoma* OR myeloma* OR tumor* OR tumour* OR sarcoma* OR osteosarcoma*) | | |
| #2 | TS=(registr* OR ((neoplas* OR cancer* OR oncolog* OR malignan* OR metasta* OR carcinom* OR adenocarcinom* OR adenoma* OR leukemia* OR leukaemia* OR lymphoma* OR myeloma* OR tumor* OR tumour* OR sarcoma* OR osteosarcoma*) NEAR/7 (incidence OR prevalence OR burden OR epidemiol*)) OR ((neoplas* OR cancer* OR oncolog* OR tumor* OR tumour* OR oncolog* OR national* OR region* OR provinc* OR institution* OR hospital OR hospitals OR facilit*) NEAR/7 (data OR database* OR report*))) | | |
| #3 | (TS=(Pakistan*) OR CU=(pakistan*)) | | |
| **DATABASE** | **Cochrane Library: CENTRAL** | | |
| **Date** | 06/21/2023 | | |
| **Results** | 30 | | |
| **Strategy** | #1 AND #2 AND #3 | | |
| #1 | (neoplas* OR cancer* OR oncolog* OR malignan* OR metasta* OR carcinom* OR adenocarcinom* OR adenom* OR leukemia* OR leukaemia* OR lymphoma* OR myeloma* OR tumor* OR tumour* OR sarcoma* OR osteosarcoma*):ti,ab,kw | | |
| #2 | (registr* OR ((neoplas* OR cancer* OR oncolog* OR malignan* OR metasta* OR carcinom* OR adenocarcinom* OR adenoma* OR leukemia* OR leukaemia* OR lymphoma* OR myeloma* OR tumor* OR tumour* OR sarcoma* OR osteosarcoma*) NEAR/7 (incidence OR prevalence OR burden OR epidemiol*)) OR ((neoplas* OR cancer* OR oncolog* OR tumor* OR tumour* OR national* OR region* OR provinc* OR institution* OR hospital OR hospitals OR facilit*) NEAR/7 (data OR database* OR report*))):ti,ab,kw | | |
| #3 | (Pakistan*):ti,ab,kw | | |
| **DATABASE** | **Pakmedinet** | | |
| **Date** | 06/21/2023 | | |
| **Results** | 30 | | |
| **Strategy** | 250 | | |
|  | neoplasm | registry | neoplasm AND registry |
|  | neoplasms | registry | neoplasms AND registry |
|  | cancer | registry | cancer AND registry |
|  | cancers | registry | cancers AND registry |
|  | oncology | registry | oncology AND registry |
|  | oncologic | registry | oncologic AND registry |
|  | malignancy | registry | malignancy AND registry |
|  | malignancies | registry | malignancies AND registry |
|  | metastasis | registry | metastasis AND registry |
|  | metastases | registry | metastases AND registry |
|  | carcinoma | registry | carcinoma AND registry |
|  | carcinomas | registry | carcinomas AND registry |
|  | adenocarcinoma | registry | adenocarcinoma AND registry |
|  | adenocarcinomas | registry | adenocarcinomas AND registry |
|  | adenoma | registry | adenoma AND registry |
|  | adenomas | registry | adenomas AND registry |
|  | leukemia | registry | leukemia AND registry |
|  | leukaemia | registry | leukaemia AND registry |
|  | lymphoma | registry | lymphoma AND registry |
|  | lymphomas | registry | lymphomas AND registry |
|  | myeloma | registry | myeloma AND registry |
|  | myelomas | registry | myelomas AND registry |
|  | tumor | registry | tumor AND registry |
|  | tumors | registry | tumors AND registry |
|  | tumour | registry | tumour AND registry |
|  | tumours | registry | tumours AND registry |
|  | sarcoma | registry | sarcoma AND registry |
|  | sarcomas | registry | sarcomas AND registry |
|  | osteosarcoma | registry | osteosarcoma AND registry |
|  | osteosarcomas | registry | osteosarcomas AND registry |
|  | neoplasm | registries | neoplasm AND registries |
|  | neoplasms | registries | neoplasms AND registries |
|  | cancer | registries | cancer AND registries |
|  | cancers | registries | cancers AND registries |
|  | oncology | registries | oncology AND registries |
|  | oncologic | registries | oncologic AND registries |
|  | malignancy | registries | malignancy AND registries |
|  | malignancies | registries | malignancies AND registries |
|  | metastasis | registries | metastasis AND registries |
|  | metastases | registries | metastases AND registries |
|  | carcinoma | registries | carcinoma AND registries |
|  | carcinomas | registries | carcinomas AND registries |
|  | adenocarcinoma | registries | adenocarcinoma AND registries |
|  | adenocarcinomas | registries | adenocarcinomas AND registries |
|  | adenoma | registries | adenoma AND registries |
|  | adenomas | registries | adenomas AND registries |
|  | leukemia | registries | leukemia AND registries |
|  | leukaemia | registries | leukaemia AND registries |
|  | lymphoma | registries | lymphoma AND registries |
|  | lymphomas | registries | lymphomas AND registries |
|  | myeloma | registries | myeloma AND registries |
|  | myelomas | registries | myelomas AND registries |
|  | tumor | registries | tumor AND registries |
|  | tumors | registries | tumors AND registries |
|  | tumour | registries | tumour AND registries |
|  | tumours | registries | tumours AND registries |
|  | sarcoma | registries | sarcoma AND registries |
|  | sarcomas | registries | sarcomas AND registries |
|  | osteosarcomas | registries | osteosarcomas AND registries |
|  | osteosarcomas | registries | osteosarcomas AND registries |
|  | cancer | registration | cancer AND registration |
|  | cancers | registration | cancers AND registration |
|  | neoplasm | registration | neoplasm AND registration |
|  | neoplasms | registration | neoplasms AND registration |
|  | oncology | registration | oncology AND registration |
|  | oncologic | registration | oncologic AND registration |
|  | malignancy | registration | malignancy AND registration |
|  | malignancies | registration | malignancies AND registration |
|  | metastasis | registration | metastasis AND registration |
|  | metastases | registration | metastases AND registration |
|  | carcinoma | registration | carcinoma AND registration |
|  | carcinomas | registration | carcinomas AND registration |
|  | adenocarcinoma | registration | adenocarcinoma AND registration |
|  | adenocarcinomas | registration | adenocarcinomas AND registration |
|  | adenoma | registration | adenoma AND registration |
|  | adenomas | registration | adenomas AND registration |
|  | leukemia | registration | leukemia AND registration |
|  | leukaemia | registration | leukaemia AND registration |
|  | lymphoma | registration | lymphoma AND registration |
|  | lymphomas | registration | lymphomas AND registration |
|  | myeloma | registration | myeloma AND registration |
|  | myelomas | registration | myelomas AND registration |
|  | tumor | registration | tumor AND registration |
|  | tumors | registration | tumors AND registration |
|  | tumour | registration | tumour AND registration |
|  | tumours | registration | tumours AND registration |
|  | sarcoma | registration | sarcoma AND registration |
|  | sarcomas | registration | sarcomas AND registration |
|  | osteosarcoma | registration | osteosarcoma AND registration |
|  | cancer | database | cancer AND database |
|  | cancers | database | cancers AND database |
|  | neoplasm | database | neoplasm AND database |
|  | neoplasms | database | neoplasms AND database |
|  | oncology | database | oncology AND database |
|  | oncologic | database | oncologic AND database |
|  | tumor | database | tumor AND database |
|  | tumors | database | tumors AND database |
|  | tumour | database | tumour AND database |
|  | tumours | database | tumours AND database |
|  | national | database | national AND database |
|  | region | database | region AND database |
|  | regional | database | regional AND database |
|  | province | database | province AND database |
|  | provincial | database | provincial AND database |
|  | institution | database | institution AND database |
|  | institutions | database | institutions AND database |
|  | institutional | database | institutional AND database |
|  | hospital | database | hospital AND database |
|  | hospitals | database | hospitals AND database |
|  | facility | database | facility AND database |
|  | facilities | database | facilities AND database |
|  | cancer | reporting | cancer AND reporting |
|  | cancers | reporting | cancers AND reporting |
|  | neoplasm | reporting | neoplasm AND reporting |
|  | neoplasms | reporting | neoplasms AND reporting |
|  | oncology | reporting | oncology AND reporting |
|  | oncologic | reporting | oncologic AND reporting |
|  | tumor | reporting | tumor AND reporting |
|  | tumors | reporting | tumors AND reporting |
|  | tumour | reporting | tumour AND reporting |
|  | tumours | reporting | tumours AND reporting |
|  | national | reporting | national AND reporting |
|  | region | reporting | region AND reporting |
|  | regional | reporting | regional AND reporting |
|  | province | reporting | province AND reporting |
|  | provincial | reporting | provincial AND reporting |
|  | institution | reporting | institution AND reporting |
|  | institutions | reporting | institutions AND reporting |
|  | institutional | reporting | institutional AND reporting |
|  | hospital | reporting | hospital AND reporting |
|  | hospitals | reporting | hospitals AND reporting |
|  | facility | reporting | facility AND reporting |
|  | facilities | reporting | facilities AND reporting |

**Supplementary Table 3: List of extracted variables from each study.**

| **Publication-Specific Variables**  * For articles reporting data from a registry, we extracted the following variables: | **Registry-Specific Variables** |
| --- | --- |
| Author list  Year of publication  Years of data reported  Journal name in which the study was published  Study design  Number of patients included  Objective of the study as outlined by the author | Name  Establishment and duration  Main purpose  Geographical area  The type of registry (e.g., hospital-level, city-level, regional)  Names and number of contributing sites and centers  Data sources (such as medical records, lab tests, and pathology labs)  Inclusion criteria for patient inclusion in the registry  Data collection methods  Follow-up mechanisms (including frequency and methods)  Who collects the data?  Specific data elements gathered  Whether inclusion in the registry is mandatory or voluntary  Feedback mechanisms  Quality control methods  Real-time data collection status  Funding sources  Incentives and barriers  Any additional operational features identified during the data extraction process |

**Supplementary Table 4: Summary of studies included in the scoping review.**

| **Author** | **Year of publication** | **Registry** | **Years of data reported** | **Journal of publication** | **Study design** | **Number of participants** | **Aim/Objective** |
| --- | --- | --- | --- | --- | --- | --- | --- |
| Ahmad et al. (1) | 1991 | Armed Forces Institute of Pathology Rawalpindi Tumor Registry | 1977-1988 | Journal of the Pakistan Medical Association | Cross sectional study | 14,018 | Epidemiology of cancer in patients |
| Ahmed et al (2) | 2015 | Armed Forces Institute of Pathology Rawalpindi Tumor Registry | 2012-2013 | Pakistan Armed Forces Medical Journal | Retrospective cohort | 541 | Analyse the clinicopathological spectrum of urothelial carcinoma of urinary bladder. |
| Akhtar et al. (3) | 2012 | Armed Forces Institute of Pathology Rawalpindi Tumor Registry | 2006-2010 | Annals of Pakistan Institute of Medical Sciences | Cross sectional study | 240 | Epidemiology of malignant jaw tumors presenting at AFIP |
| Ali et al. (4) | 2023 | Cancer registry at NIMRA cancer hospital | 2015-2021 | Dr. Sulaiman Al Habib Medical Journal | Cross sectional study | 161,191 | Epidemiology - Cancer trends at a hospital |
| Anwar et al. (5) | 2022 | Armed Forces Institute of Pathology Rawalpindi Tumor Registry | 2009-2018 | Pakistan Armed Forces Medical Journal | Cross sectional study | 37793 | Frequency and distribution of male genital tract tumours and urinary system malignancies and their changing trends in Pakistan |
| Anwer et al. (6) | 2017 | Shaukat Khanum Memorial Cancer Hospital and Research Centre (SKMCH & RC)'s Hospital Cancer Registry | 2004-2014 | Journal of Cancer and Allied Specialties | Cross sectional study | 5027 | Epidemiology of head and neck cancers in Pakistan across a 10-year period |
| Ashfaq et al. (7) | 2014 | Armed Forces Institute of Pathology Rawalpindi Tumor Registry | 2008-2011 | Journal of the College of Physicians and Surgeons Pakistan | Cross sectional study | 110 | Epidemiological data on submandibular gland involvement in early oral cavity tumors using AFIP registry data. |
| Ashfaq et al. (8) | 2012 | Armed Forces Institute of Pathology Rawalpindi Tumor Registry | 2008-2011 | Journal of Islamic International Medical College | Cross sectional study | 113 | Epidemiological data on oral cavity tumors using AFIP registry data. |
| Asif et al. (9) | 2010 | Armed Forces Institute of Pathology Rawalpindi Tumor Registry | 2005-2007 | Asian Pacific Journal of Cancer Prevention | Cross sectional study | 235 | Determine the demographics of BCC and status of margin clearance in our population. |
| Aziz et al. (10) | 2003 | Tumor Registry at Jinnah Hospital | 1997-2001 | Journal of the Pakistan Medical Association | Cross sectional study | 5100 | Frequencies of different cancers presented to Jinnah Hospital |
| Badar et al. (11) | 2022 | Karachi Cancer Registry; Punjab Cancer Registry; Shaukat Khanum Memorial Cancer Hospital and Research Centre registry; Pakistan Atomic Energy Commission's (PAEC) cancer registry; National Cancer Registry | - | Journal of Cancer and Allied Specialties | Editorial | - | Descriptive study – no new data  to discuss current Pakistan cancer registry publications and explain the need for expanding them |
| Badar et al. (12) | 2019 | Punjab Cancer Registry | - | Journal of Ayub Medical College | Letter to the Editor | - | Descriptive study – no new data |
| Badar et al. (13) | 2015 | Shaukat Khanum Memorial Cancer Hospital and Research Centre (SKMCH & RC)'s Hospital Cancer Registry | 2011-2012 | Journal of Ayub Medical College Abbottabad | Cross sectional study | 669 | Epidemiology and cancer incidence |
| Badar et al. (14) | 2015 | Shaukat Khanum Memorial Cancer Hospital and Research Centre (SKMCH & RC)'s Hospital Cancer Registry | 2008-2012 | Journal of the College of Physicians and Surgeons Pakistan | Cross sectional study | 4411 | Epidemiology, cancer incidence |
| Badar et al. (15) | 2011 | Shaukat Khanum Memorial Cancer Hospital and Research Centre (SKMCH & RC)'s Hospital Cancer Registry | - | Asian Pacific Journal of Cancer Prevention | Commentary | - | Descriptive study – no new data |
| Badar et al. (16) | 2015 | Shaukat Khanum Memorial Cancer Hospital and Research Centre (SKMCH & RC)'s Hospital Cancer Registry | 1994-2012 | Journal of the College of Physicians and Surgeons Pakistan | Cross sectional study | 58761 | Frequency distribution of cancer cases |
| Badar et al. (17) | 2017 | Punjab Cancer Registry (PCR) | 2010-2012 | BMJ Open | Cross sectional study | 15825 | Cancer incidence by age group for the Lahore district population |
| Badar et al. (18) | 2022 | Punjab Cancer Registry (PCR) | 2010-2019 | BMJ Open | Cross sectional study | 58394 | Cancer incidence rates over 10 years (2010–2019), in Lahore, Pakistan. |
| Badar et al. (19) | 2020 | Punjab Cancer Registry (PCR) | 2010-2015 | Journal of the College of Physician and Surgeons Pakistan | Cross sectional study | 32977 | Cancer incidence rates in Lahore; according to age group, age-specific incidence rates for cancers. |
| Badar et al. (20) | 2016 | Punjab Cancer Registry (PCR) | 2010-2012 | BMJ Open | Cross sectional study | 15 840 | Population-level cancer estimates for the Lahore, Age-Standardized Incidence Rates (ASIR) per 100,000 population by gender and cancer site, mortality rates |
| Badar et al. (21) | 2009 | Shaukat Khanum Memorial Cancer Hospital and Research Centre (SKMCH & RC)'s Hospital Cancer Registry | 1994-2004 | Asian Pacific Journal of Cancer Prevention | Cross sectional study | 607 | Epidemiologic features of urinary bladder cancer cases presenting at a cancer hospital. |
| Basbous et al. (22) | 2020 | Karachi Cancer Registry  Aga Khan University Cancer Registry | - | Cancer Epidemiology | Literature review | - | Descriptive study – no new data  snapshot of successes and barriers in childhood cancer treatment, focusing on five countries from the Middle East, North Africa, and West/Central Asia region. |
| Bhurgri et al. (23) | 2011 | Karachi cancer registry | 1995-1999 | IACR Scientific Publications | Cross sectional study | 677 | Survival statistics of head and neck cancers from 1995-1999 in Karachi |
| Bhurgri et al. (24) | 2004 | Karachi Cancer Registry | 1995-2002 | Asian Pacific Journal of Cancer Prevention | Commentary | 1,724,915  (Karachi population) | Incidence of various cancer types, common risk factors reported. |
| Bhurgri et al. (25) | 2005 | Karachi Cancer Registry | 1995-2002 | Asian Pacific Journal of Cancer Prevention | Cross sectional study | 2253 | Trends of oral cancer, age-and socioeconomic profile over time in Karachi |
| Bhurgri et al. (26) | 2003 | Karachi Cancer Registry | 1995-2001 | Asian Pacific Journal of Cancer Prevention | Cross sectional study | 1482 | Demographics, potential risk factors, ASIR of cancer of the oral cavity and pharynx in Karachi. |
| Bhurgri et al. (27) | 2005 | Karachi Cancer Registry; Aga Khan University Pathology-based Cancer Registry | 1998-2002 | Asian Pacific Journal of Cancer Prevention | Cross sectional study | - | Trends, incidence, ASIR of cancers in Hyderabad, Karachi. |
| Bhurgri et al. (28) | 2004 | Karachi Cancer Registry | 1998-2002 | Asian Pacific Journal of Cancer Prevention | Cross sectional study | 217 | Report epidemiological features of rhabdomyosarcoma from the Karachi Cancer Registry |
| Bhurgri et al. (29) | 1999 | Karachi Cancer Registry | 1995-1996 | Journal of the Pakistan Medical Association | Cross sectional study | Estimated 2.85 million (Karachi population) | Cancer pattern by site, age, sex in Karachi |
| Bhurgri et al. (30) | 2006 | Karachi Cancer Registry | 1995-2002 | Asian Pacific Journal of Cancer Prevention | Cross sectional study | 1,724,915  (Karachi population) | Epidemiological characteristics, incidence and time trends of head and neck cancers |
| Bhurgri et al. (31) | 2006 | Karachi Cancer Registry | 1995-2005 | Asian Pacific Journal of Cancer Prevention | Cross sectional study | 1,724,915  (Karachi population) | Epidemiological characteristics, incidence and time trends of lung cancers by site, age, sex, grades. |
| Bhurgri et al. (32) | 2003 | Karachi Cancer Registry | 1998-2002 | Asian Pacific Journal of Cancer Prevention | Cross sectional study | 242 | Epidemiological characteristics, incidence and time trends of ocular malignancies by site, age, sex, grades. |
| Bhurgri et al. (33) | 2011 | Karachi Cancer Registry | 1995-2002 | Asian Pacific Journal of Cancer Prevention | Cross sectional study | 151 | Epidemiological characteristics, incidence and time trends of colorectal carcinoma by site, age, sex, grades. |
| Bhurgri et al. (34) | 2004 | Karachi Cancer Registry | 1998-2002 | Asian Pacific Journal of Cancer Prevention | Cross sectional study | 101 | Epidemiological characteristics, incidence and time trends of retinoblastoma by site, age, sex, grades. |
| Bhurgri et al.  (35) | 2005 | Karachi Cancer Registry | 1995-2002 | Asian Pacific Journal of Cancer Prevention | Cross sectional study | 429 | Epidemiological characteristics, incidence and time trends of Non-Hodgkin’s Lymphoma by site, age, sex, grades. |
| Bhurgri et al. (36) | 2006 | Karachi Cancer Registry (KCR); Aga Khan University Pathology-Based Cancer Registry (APCR) | 2000-2002 | Asian Pacific Journal of Cancer Prevention | Cross sectional study | Larkana population | Epidemiological characteristics, incidence and time trends of by site, age, sex, grades of Larkana. |
| Bhurgri et al. (37) | 2002 | Karachi Cancer Registry | 1995-2001 | Journal of the Pakistan Medical Association | Cross sectional study | 1,658,638  (Karachi population as of 1998) | Comparison of two registry data sets from Karachi, one from Karachi South, and one from Karachi Division to identify changes in registration numbers and incidence of cancers in Karachi. |
| Bhurgri et al. (38) | 2002 | Karachi Cancer Registry | 1998-1999 | Journal of the Pakistan Medical Association | Cross sectional study | 7396 | First published cancer incidence data for Karachi. |
| Bhurgri et al. (39) | 2000 | Karachi Cancer Registry | 1995-1997 | International Journal of Cancer | Cross sectional study | 4268 | Report incidence data from the KCR |
| Bhurgri et al. (40) | 2011 | Karachi Cancer Registry | 1995-2002 | Asian Pacific Journal of Cancer Prevention | Cross sectional study | 321 | Epidemiological characteristics, incidence and time trends of primary CNS malignancies by site, age, sex, grades. |
| Bhurgri et al. (41) | 2008 | Karachi Cancer Registry | 1995-1997 | Asian Pacific Journal of Cancer Prevention | Cross sectional study | 96 | Epidemiological characteristics, incidence and time trends of soft tissue sarcoma |
| Bhurgri et al. (42) | 2004 | Karachi Cancer Registry | 1995-2002 | Journal of the Pakistan Medical Association | Cross sectional study | 1,724,915  (Karachi population) | Epidemiological characteristics, incidence and time trends of esophagus cancer site, age, sex, grades. |
| Bhurgri et al. (43) | 2007 | Karachi Cancer Registry | 1995-1997 | Asian Pacific Journal of Cancer Prevention | Cross sectional study | 709 | Epidemiological characteristics, incidence and time trends of breast cancer by site, age, sex, grades. |
| Bhurgri et al. (44) | 2009 | Karachi Cancer Registry | 1995-2002 | Asian Pacific Journal of Cancer Prevention | Cross sectional study | 282 | Epidemiological characteristics, incidence and time trends of prostate cancer by site, age, sex, grades. |
| Bhurgri et al. (45) | 2004 | Karachi Cancer Registry | 1998-2002 | Journal of the Pakistan Medical Association | Cross sectional study | 10 | Epidemiological characteristics, incidence and time trends of ocular rhabdomyosarcoma by site, age, sex, grades. |
| Bhurgri et al. (46) | 2007 | Karachi Cancer Registry | 1995-1997 | Asian Pacific Journal of Cancer Prevention | Cross sectional study | 66 | Epidemiological characteristics, incidence and time trends of cancer corpus uteri by site, age, sex, grades. |
| Bhurgri et al. (47) | 2007 | Karachi Cancer Registry | 1995-1997 | Asian Pacific Journal of Cancer Prevention | Cross sectional study | 74 | Epidemiological characteristics, incidence and time trends of cervical cancer by site, age, sex, grades. |
| Bhurgri et al. (48) | 2008 | Karachi Cancer Registry | 1995-2002 | Asian Pacific Journal of Cancer Prevention | Cross sectional study | 233 | Epidemiological characteristics, incidence and time trends of cervical by site, age, sex, grades. |
| Bhurgri et al. (49) | 2009 | Karachi Cancer Registry | 1995-2002 | Asian Pacific Journal of Cancer Prevention | Cross sectional study | 335 | Epidemiological characteristics, incidence and time trends of gastric cancer by site, age, sex, grades. |
| Bhurgri et al. (50) | 2002 | Karachi Cancer Registry | 1998-1999 | Journal of the Pakistan Medical Association | Cross sectional study | 1288 | Epidemiological characteristics, incidence and time trends of cancer in Quetta. |
| Bhurgri et al. (51) | 1998 | Karachi Cancer Registry | 1995-1996 | Journal of the Pakistan Medical Association | Cross sectional study | 233 | Epidemiological characteristics, incidence and time trends of oral cancers by site, age, sex, grades. |
| Bhurgri et al. (52) | 2011 | Karachi Cancer Registry | 1995-2002 | Asian Pacific Journal of Cancer Prevention | Cross sectional study | 337 | Epidemiological characteristics, incidence and time trends of ovarian cancer by site, age, sex, grades. |
| Bhurgri et al. (53) | 2009 | Karachi Cancer Registry | 1995-1997 | Asian Pacific Journal of Cancer Prevention | Cross sectional study | 68 | Epidemiological characteristics, incidence and time trends of primary malignancies of bone and cartilage by site, age, sex, grades. |
| Bhurgri et al. (54) | 2003 | Karachi Cancer Registry | 1995-2005 | Indian Journal of Gastroenterology | Prevalence study | Karachi population | Epidemiological study to report esophagus cancer burden in Karachi and Quetta. |
| Chughtai et al. (55) | 2023 | Karachi Cancer Registry (KCR); Dow Cancer Registry; Punjab Cancer Registry (PCR); Shaukat Khanum Cancer Registry; Pakistan Atomic Energy Cancer Registries (PAEC) | 1995-2022 | BMC Public Health | Systematic review | - | Estimated the burden (age specific incidence rates and age standardized incidence rates - ASIR) of cervical cancer in Pakistan using published data - 13 studies. |
| Cancela et al. (56) | 2009 | Karachi Cancer Registry | 1998-2002 | Head and Neck | Cross sectional study | 901 | Incidence of oral cavity cancers compared in developed vs developing countries using registry data from multiple countries. |
| Fadoo et al. (57) | 2010 | Aga Khan University registry | 2010-2019 | Journal of Pediatric Hematology/Oncology | Retrospective cohort study | 2,694 | Compilation and analysis of pediatric cancer data at AKU registry |
| Hafeez et al. (58) | 2020 | Punjab Cancer Registry | 2010-2016 | Journal of Bioresource Management | Cross sectional study | 13,981  *Also included data from various hospitals not a part of PCR | Trends in major cancers (breast, prostate, head and neck, cervical, and colon) prevalence in Pakistan. |
| Hassan et al. (59) | 2022 | Karachi Cancer Registry (KCR); Punjab Cancer Registry (PCR) | - | Journal of Cancer Policy | Literature Review | - | Descriptive study – no new data  Describe the existing status for cancer registries and research in Pakistan and suggest recommendations |
| Hayat et al. (60) | 2018 | Tumor registry at Allied Hospital, Faisalabad | 2002-2011 | Indo American Journal of Pharmaceutical Sciences | Cross sectional study | 32718 | Epidemiological data and tumor trends |
| Hussain et al. (61) | 2008 | Institute of Radiotherapy and Nuclear Medicine, Peshawar, Pakistan registry | 1995-2001 | European Journal of Cancer Care | Cross sectional study | 2134 | Provide baseline data on frequency, morphological types, gender and age distribution of breast cancer in North-West Frontier Province and Federally Administered Tribal Areas of Pakistan. |
| Hussain et al. (62) | 2016 | Central Nervous System Tumour registry (Combined Military Hospital, Rawalpindi) | 2011-2015 | Pakistan Journal of Neurological Surgery | Retrospective observational study | 653 | Epidemiological data and tumor trends of CNS tumors in the registry. |
| Ikram et al. (63) | 2023 | Punjab Cancer Registry (PCR); Karachi Cancer Registry (KCR); Pakistan Atomic Energy Commission (PAEC) Cancer Registry; Armed Forces Institute of Pathology (AFIP) Tumor Registry; Nishtar Medical University Hospital Multan (NMH) registry; Shifa International Hospital, Islamabad (SIH) registry | 2015-2019 | Journal of the College of Physicians and Surgeons Pakistan | Cross sectional study | 269,707 | Merged and analysed cancer registration data received from major functional cancer registries in various parts of Pakistan |
| Jahangir et al. (64) | 2021 | Shaukat Khanum Memorial Cancer Hospital and Research Centre (SKMCH & RC)'s Hospital Cancer Registry | 2004-2014 | Cureus | Cross sectional study | 78 | Assess the expression of CD117 (c-KIT) in phyllodes tumor of the breast and its relationship with morphology and clinical outcome using registry data |
| Jamal et al. (65) | 2014 | Armed Forces Institute of Pathology Rawalpindi Tumor Registry | 2002-2011 | Journal of the Pakistan Medical Association | Cross sectional study | 32718 | Pattern of tumor trends using registry data. |
| Jamal et al. (66) | 2020 | Armed Forces Institute of Pathology Rawalpindi Tumor Registry | 2009-2018 | Pakistan Armed Forces Medical Journal | Cross sectional study | 1873 | Epidemiology and clinicopathological pattern of malignant tumours |
| Jamal et al. (67) | 2020 | Armed Forces Institute of Pathology Rawalpindi Tumor Registry | 2009-2018 | Journal of the Pakistan Medical Association | Cross sectional study | 1279 | Epidemiology and clinicopathological pattern of childhood tumours |
| Jamal et al. (68) | 2005 | Armed Forces Institute of Pathology Rawalpindi Tumor Registry | 1992-2001 | Asian Pacific Journal of Cancer Prevention | Cross sectional study | 2279 | Epidemiological pattern of gastrointestinal malignancies in northern Pakistan |
| Jamal et al. (69) | 2006 | Armed Forces Institute of Pathology Rawalpindi Tumor Registry | 1992-2001 | Asian Pacific Journal of Cancer Prevention | Cross sectional study | 922 | Epidemiological data of childhood tumours |
| Jamal et al. (70) | 2006 | Armed Forces Institute of Pathology Rawalpindi Tumor Registry | 1992-2001 | Annals of Saudi Medicine | Cross sectional study | 968 | Epidemiological data of gynaecological tumours |
| Jamal et al. (71) | 2006 | Armed Forces Institute of Pathology Rawalpindi Tumor Registry | 1992-2001 | Journal of the Pakistan Medical Association | Cross sectional study | 21,168 | Epidemiological data on malignant tumours from registry data. |
| Jamal et al. (72) | 2005 | Armed Forces Institute of Pathology Rawalpindi Tumor Registry | 1992-2001 | Pakistan Journal of Pathology | Cross sectional study | 430 | Epidemiological data on malignant brain tumours from registry data. |
| Janjua et al. (73) | 2013 | Armed Forces Institute of Pathology Rawalpindi Tumor Registry | 2008-2010 | Journal of the College of Physicians and Surgeons Pakistan | Cross sectional study | 319 | Epidemiological data on head and neck malignant tumours from registry data. |
| Javed et al. (74) | 2006 | AFIP Tumours Registry; Karachi cancer registry; JPMC Karachi; | - | Indian Journal of Medical and Paediatric Oncology | Report – literature review | - | Descriptive study – no new data  Reported incidence rates from other primary studies based on registry data. |
| Kayani et al. (75) | 2011 | Armed Forces Institute of Pathology Rawalpindi Tumor Registry | 1987-2008 | Asian Pacific Journal of Cancer Prevention | Cross sectional study | 234 | Epidemiological data on non-cutaneous malignant melanoma. |
| Khadim et al. (76) | 2011 | Armed Forces Institute of Pathology Rawalpindi Tumor Registry | 2008-2010 | Asian Pacific Journal of Cancer Prevention | Cross sectional study | 130 | Evaluate the role of immunohistochemistry  in metastatic liver disease using registry data |
| Mahmood et al. (77) | 2018 | Shaukat Khanum Memorial Cancer Hospital and Research Centre (SKMCH & RC)'s Hospital Cancer Registry | 2017 | Not published in journal | Annual report of the registry | 6,439 | Reported cancer statistics for patients registered. |
| Mamoon et al. (78) | 2009 | Armed Forces Institute of Pathology Rawalpindi Tumor Registry | 2005-2008 | Asian Pacific Journal of Cancer Prevention | Cross sectional study | 1644 | Epidemiology of early onset breast cancer (,30 years) in Pakistani population |
| Masood et al. (79) | 2018 | Pakistan Atomic Energy Commission's (PAEC) Cancer Registry | 1984-2014 | Asian Pacific Journal of Cancer Prevention | Cross sectional study | - | Trends and patterns of most common cancers from Lahore, Pakistan |
| Munawar et al. (80) | 2023 | Shaukat Khanum Memorial Cancer Hospital and Research Centre (SKMCH & RC)'s Hospital Cancer Registry | 2013-2021 | Journal Of Cancer & Allied Specialties | Cross sectional study | 32 | Present the first account of the clinical outcomes of EGFR-TKIs in EGFR-mutant lung adenocarcinoma among Pakistani Asians. |
| Mushtaq et al. (81) | 2008 | Armed Forces Institute of Pathology Rawalpindi Tumor Registry | 2005 | Asian Pacific Journal of Cancer Prevention | Cross sectional study | 246 | Identify the spectrum of malignant lymphoma in Pakistan, according to the WHO classification |
| Nawaz et al. (82) | 2015 | Punjab Institute of Nuclear Medicine (PINUM) Cancer Registry | 2010-2011 | International Journal of Applied Sciences and Biotechnology | Prevalence study | 690 | Epidemiology of lymphoma cancer registry record from Faisalabad. |
| Pervez (83) | 2012 | Karachi cancer registry (KCR); Shaukat Khanum Memorial Hospital (SKMH &RC), Lahore cancer registry; AFIP, Rawalpindi Pakistan cancer registry | - | International Journal of Molecular and Cellular Medicine | Short communication, literature review | - | Descriptive study – no new data  Discuss the burden of non-Hodgkin lymphoma in Pakistan |
| Pervez et al. (84) | 2020 | Karachi Cancer Registry (KCR) | 2017-2019 | Asian Pacific Journal of Cancer Prevention | Cross sectional study | 33,309 | Estimate the cancer incidence by age group and gender for the population of Karachi Division. |
| Pervez et al. (85) | 2023 | Karachi Cancer Registry (KCR) | 2017-2021 | Journal of the College of physicians and Surgeons Pakistan | Cross sectional study | 65,886 | Epidemiologic data of all malignancies by age group and gender for the Karachi population. |
| Qureshi et al. (86) | 2020 | Dow Cancer Registry | 2010-2019 | Pakistan Journal of Medical Sciences | Cross sectional study | 22,858 | Epidemiological data on cancer burden in Karachi |
| Qureshi et al. (87) | 2016 | Dow Cancer Registry | 2010-2015 | Cancer Epidemiology | Cross sectional study | 13,508 | Epidemiological data on cancer burden and comparison of cancer patterns to various Asian populations. |
| Qureshi et al. (88) | 2020 | Dow Cancer Registry | 2010-2019 | Journal of Taibah University Medical Sciences | Cross sectional study | 22,858 | To provide an overview of lip and oral cavity cancer in Karachi. |
| Rahman et al. (89) | 2008 | Armed Forces Institute of Pathology Rawalpindi Tumor Registry | 1994-2003 | Haematology/Oncology and Stem Cell Therapy | Cross sectional study | 21168 | To analyse minor salivary gland tumors in the population in Northern Pakistan (AFIP) |
| Sarwar et al. (90) | 2006 | Shaukat Khanum Memorial Cancer Hospital and Research Centre (SKMCH & RC)'s Hospital Cancer Registry | 1994-2003 | Asian Pacific Journal of Cancer Prevention | Cross sectional study | 544 | To analyse malignant epithelial ovarian cancer at a tertiary care cancer hospital in Pakistan. |
| Shafqat et al. (91) | 2022 | Patel hospital cancer registry database | 2008-2018 | Journal of the Pakistan Medical Association | Retrospective cohort study | 450 | Assess the recurrence and survival in OSCC patients, and to compare them between young and old age groups |
| Sheikh et al. (92) | 2022 | Karachi Cancer Registry; Punjab Cancer Registry | - | Journal of Thoracic Oncology | Editorial | - | Descriptive study – no new data  Discusses the burden of lung cancer using reported data in literature. |
| Shamim et al. (93) | 2021 | Aga Khan University, Department of anaesthesiology - registry database for perioperative anaesthetic management in head and neck cancer surgery. | 2018-2019 | Cureus | Retrospective cohort study | 325 | To evaluate the use of video laryngoscope in patients for head and neck cancer surgery with regards to success, failure, and intubation-related complications. |
| Siddiqui et al. (94) | 2006 | Shaukat Khanum Memorial Cancer Hospital and Research Centre (SKMCH & RC)'s Hospital Cancer Registry | 1995-2003 | Asian Pacific Journal of Cancer Prevention | Cross sectional study | 658 | to study the clinical and epidemiological profile of Hodgkin's lymphoma (HL) in Pakistan |
| Sultan et al. (95) | 2018 | Liaquat National Hospital and Medical College hematologic tumour registry | 2012-2016 | Journal of Laboratory Physicians | Cross sectional study | 366 | Report a 4 years' institutional-based haematological tumor registry data from a tertiary care hospital in Karachi. |
| Tufail et al. (96) | 2023 | Pakistan Atomic Energy Commission's (PAEC) Cancer Registry | 2015-2019 | Journal of Epidemiology and Global Health | Cross sectional study | 40,797 | Cancer statistics in Pakistan to summarize the status of cancer prevalence, types, and other relevant data specific to Pakistan |
| Umer et al. (97) | 2014 | Aga Khan University Cancer Registry | 1994-2011 | Journal of the Pakistan Medical Association | Cross sectional study | 9 | Institutional experience of paediatric patients having undergone skeletal reconstruction after limb salvage |
| Umer at al. (98) | 2021 | Aga Khan University Cancer Registry | 2017-2018 | Journal of the Pakistan Medical Association | Prevalence study | 119 | Study the frequency of the thigh, hip and groin soft tissue sarcomas. |
| Wickramasinghe et al. (99) | 2016 | Karachi Cancer Registry | - | Asia-Pacific Journal of Clinical Oncology | Cross sectional study | - | No new data from Pakistan registry reported.  Reported Sri Lanka cancer data and compared it with reported stats from Karachi Cancer Registry. |
| Yusuf (100) | 2013 | Punjab Cancer Registry | - | Japanese Journal of Clinical Oncology | Literature review | - | Descriptive study - no new data from Pakistan registry reported. |
| Zeb et al. (101) | 2006 | Cancer registry of Institute of Radiotherapy and Nuclear Medicine (IRNUM) Peshawar | 2000-2004 | Asian Pacific Journal of Cancer Prevention | Cross sectional study | 1150 | Cancer incidence among low socioeconomic area in Pakistan |
| Zeb at al. (102) | 2008 | Cancer registry of Institute of Radiotherapy and Nuclear Medicine (IRNUM) Peshawar | 2000-2004 | Journal of Chinese Medical Association | Cross sectional study | 1105 | Analyse the cancer registry record of patients from the districts in Dir in order to obtain a net cancer incidence. |

*Aim/Objective refers to the objective of the study as outlined by the respective authors

**Supplementary Table 5:** **Collaborating centers for multi-institutional level registries**

| **Registry** | **Data Source** |
| --- | --- |
| Karachi Cancer Registry | **Years 1995-2017**   1. Jinnah Postgraduate Medical Centre (JPMC) 2. Civil Hospital (CH) 3. Lyari General Hospital (LGH - Lyari) 4. Lady Dufferin Hospital (LDH) 5. Sarfaraz Rafiqui Shaheed Hospital (SRSH) 6. Karachi Adventist Hospital (KAH) 7. Anklesaria Hospital (AH) 8. Memon Hospital (MH) 9. Aga Khan University Hospital (AKUH) 10. Liaquat National Hospital (LNH) 11. Baqai Institute of Oncology Medicare Hospital (BIOMH) 12. Edhi Cancer Center (ECC) 13. Ziauddin Cancer Hospital (ZCH)   **Years 2017 onwards**   1. Aga Khan University Hospital (AKUH) 2. Jinnah Postgraduate Medical Center (JPMC) 3. Dow University of Medical Sciences (DUHS) 4. Indus Hospital (IH) 5. Ziauddin University Hospital (ZUH) 6. Zainab Punjwani Hospital (ZPH) 7. Baqai Institute of Oncology Medicare Hospital (BIOMH) |
| Punjab Cancer Registry | 1. Akhtar Saeed Medical College, Lahore (ASMC) 2. Allama Iqbal Medical College (AIMC), Lahore 3. Children’s hospital (CH), Lahore 4. Children‟s Hospital (CH), Faisalabad 5. Chughtai‟s Lab (CL), Lahore 6. Combined Military Hospital (CMH), Lahore 7. Doctors Hospital (DH) 8. Excel Labs (EL) 9. Fatima Jinnah Medical College (FJMC), Lahore 10. Fatima Memorial Hospital (FMH), Lahore 11. Hameed Latif Hospital (HLH), Lahore 12. Indus Lab (IL), Lahore 13. Institute of Nuclear Medicine and Oncology, Lahore (INMOL) 14. Ittefaq Hospital (IH), Lahore 15. Jinnah Hospital (JH), Lahore 16. King Edward Medical University (KEMU) 17. Lahore General Hospital (LGH), Lahore 18. Lahore Medical and Dental College (LMDC) 19. Mughal Lab (ML) 20. Pakistan Kidney and Liver Institute and Research Center (PKLIRC), Lahore 21. Post Graduate Medical Institute (PGMI), Lahore 22. Pride Lab (PL), Lahore 23. Services Institute of Medical Sciences (SIMS), Lahore 24. Shalamar Medical & Dental College, Hospital (SMDC), Lahore 25. Shaukat Khanum Memorial Cancer Hospital and Research Center (SKMCH & RC) 26. Sheikh Zayed Hospital (SZH), Lahore 27. Social Security Hospital (SSH), Lahore 28. University Medical and Dental College (UMDC), Faisalabad |
| Pakistan Atomic Energy Commission's (PAEC) Cancer Registry | 1. Atomic Energy Medical Center (AEMC) 2. Bahawalpur Institute of Nuclear Medicine & Oncology (BINO) 3. Center for Nuclear Medicine and Radiotherapy (CENAR) Quetta 4. Institute of Nuclear Medicine, Oncology and Radiotherapy (INOR), Abbottabad 5. Institute of Radiotherapy and Nuclear Medicine (IRNUM), Peshawar 6. Institute Of Nuclear Medicine & Oncology (INMOL), Lahore 7. Karachi Institute of Radiotherapy and Nuclear Medicine (KIRAN), Karachi 8. Larkana Institute of Nuclear Medicine and Radiotherapy (LINAR) 9. Multan Institute of Nuclear Medicine and Radiotherapy (MINAR) 10. Nuclear Institute of Medicine & Radiotherapy (NIMRA), Jamshoro 11. Nuclear Medicine Oncology & Radiotherapy Institute (NORI), Islamabad 12. Punjab Institute of Nuclear Medicines (PINUM), Faisalabad 13. Gujranwala Institute of Nuclear Medicine & Radiotherapy (GINUM) 14. Bannu Institute of Nuclear Medicine Oncology and Radiotherapy, (BINOR) 15. DI Khan Institute of Nuclear Medicine and Radiotherapy (DINAR) 16. Swat Institute of Nuclear Medicine, Oncology & Radiotherapy (SINOR) 17. Nuclear Medicine Oncology and Radiotherapy Institute (NORIN) Nawabshah 18. Gilgit Institute of Nuclear Medicine, Oncology and Radiotherapy (GINOR) 19. Diagnostic Center Narowal (DCN) |

**REFERENCES**

1. Ahmad M, Khan AH, Mansoor A. The pattern of malignant tumours in northern Pakistan. JPMA The Journal of the Pakistan Medical Association. 1991;41(11):270-3.

2. Ahmed R, Hashmi SN, Din HU, Muhammad I. CLINICOPATHOLOGICAL SPECTRUM OF UROTHELIAL CARCINOMA OF THE URINARY BLADDER – A STUDY OF 541 CASES AT AFIP PAKISTAN: Multi-Tier Medical Support. Pakistan Armed Forces Medical Journal. 2015;65(4):544-7.

3. Ali Z. HISTOPATHOLOGICAL SPECTRUM OF MALIGNANT JAW TUMORS DIAGNOSED AT ARMED FORCES INSTITUTE OF PATHOLOGY (AFIP). 2012.

4. Ali F, Hussain S, Memon SA, Iqbal SS. Recently Top Trending Cancers in a Tertiary Cancer Hospital in Pakistan. Dr Sulaiman Al Habib Medical Journal. 2023;5(2):42-9.

5. Anwar M, Khadim M, Asif M, Ud Din H, Jamal S, Chaudary U. Malignant Male Genital Tract and Urinary System Tumours: Tumour Registry Data Analysis at Armed Forces Institute of Pathology, Pakistan (2009-2018). Pakistan Armed Forces Medical Journal. 2022;72:1867-70.

6. Anwer AW, Faisal M, Malik AA, Jamshed A, Hussain R, Pirzada MT, editors. HEAD AND NECK CANCER IN A DEVELOPING COUNTRY- A HOSPITAL BASED RETROSPECTIVE STUDY ACROSS 10 YEARS FROM PAKISTAN2018.

7. Ashfaq K, Ashfaq M, Ahmed A, Khan M, Azhar M. Submandibular Gland Involvement in Early Stage Oral Cavity Carcinomas: Can the Gland be left behind? Journal of the College of Physicians and Surgeons--Pakistan : JCPSP. 2014;24:565-8.

8. Ashfaq K, Ashfaq M, Hameed M, Ayub Z. Oral Cavity Tumours, A Clinical Experience in a Tertiary Care Center. Journal of Islamic International Medical College. 2012;7:44-8.

9. Asif M, Mamoon N, Ali Z, Akhtar F. Epidemiological and excision margin status of Basal cell carcinoma--three years Armed Forces Institute of Pathology experience in Pakistan. Asian Pacific journal of cancer prevention : APJCP. 2010;11(5):1421-3.

10. Aziz Z, Sana S, Saeed S, Akram M. Institution based tumor registry from Punjab: five year data based analysis. JPMA The Journal of the Pakistan Medical Association. 2003;53(8):350-3.

11. Badar F. Cancer Registration in Pakistan: a Reality Check. Journal of cancer & allied specialties. 2022;8(2):465.

12. Badar F. Setting-Up A Death Registry. Journal of Ayub Medical College, Abbottabad : JAMC. 2019;31(1):138.

13. Badar F, Mahmood S. CANCERS AMONG CHILDREN AND ADOLESCENTS AT A CANCER HOSPITAL IN PAKISTAN. Journal of Ayub Medical College, Abbottabad : JAMC. 2015;27(4):904-10.

14. Badar F, Mahmood S, Faraz R, Quader AU, Asif H, Yousaf A. Epidemiology of Breast Cancer at the Shaukat Khanum Memorial Cancer Hospital and Research Center, Lahore, Pakistan. Journal of the College of Physicians and Surgeons--Pakistan : JCPSP. 2015;25(10):738-42.

15. Badar F, Faruqui ZS, Uddin N, Trevan EA. Management of breast lesions by breast physicians in a heavily populated South asian developing country. Asian Pacific journal of cancer prevention : APJCP. 2011;12(3):827-32.

16. Badar F, Mahmood S. Hospital-based cancer profile at the Shaukat Khanum Memorial Cancer Hospital and Research Centre, Lahore, pakistan. Journal of the College of Physicians and Surgeons--Pakistan : JCPSP. 2015;25(4):259-63.

17. Badar F, Mahmood S. Epidemiology of cancers in Lahore, Pakistan, among children, adolescents and adults, 2010–2012: a cross-sectional study part 2. BMJ Open. 2017;7(12):e016559.

18. Badar F, Mahmood S. Cancer in Lahore, Pakistan, 2010-2019: an incidence study. BMJ Open. 2021;11(8):e047049.

19. Badar F, Mahmood S, Mahmood MT, Masood M, Tanvir I, Chughtai OR, et al. Cancer Epidemiology in Lahore, Pakistan - 2010-2015. Journal of the College of Physicians and Surgeons--Pakistan : JCPSP. 2020;30(2):113-22.

20. Badar F, Mahmood S, Yusuf MA, Sultan F. Epidemiology of cancers in Lahore, Pakistan, 2010-2012: a cross-sectional study. BMJ Open. 2016;6(6):e011828.

21. Badar F, Sattar A, Meerza F, Irfan N, Siddiqui N. Carcinoma of the urinary bladder in a tertiary care setting in a developing country. Asian Pacific journal of cancer prevention : APJCP. 2009;10(3):449-52.

22. Basbous M, Al-Jadiry M, Belgaumi A, Sultan I, Al-Haddad A, Jeha S, et al. Childhood cancer care in the Middle East, North Africa, and West/Central Asia: A snapshot across five countries from the POEM network. Cancer epidemiology. 2021;71(Pt B):101727.

23. Bhurgri Y. Cancer survival in South Karachi, Pakistan, 1995-1999. IARC scientific publications. 2011(162):143-6.

24. Bhurgri Y. Karachi Cancer Registry Data--implications for the National Cancer Control Program of Pakistan. Asian Pacific journal of cancer prevention : APJCP. 2004;5(1):77-82.

25. Bhurgri Y. Cancer of the oral cavity - trends in Karachi South (1995-2002). Asian Pacific journal of cancer prevention : APJCP. 2005;6(1):22-6.

26. Bhurgri Y, Bhurgri A, Hussainy A, Usman A, Faridi N, Malik J, et al. Cancer of the Oral Cavity and Pharynx in Karachi – Identification of Potential Risk Factors. Asian Pacific journal of cancer prevention : APJCP. 2003;4:125-30.

27. Bhurgri Y, Bhurgri A, Pervez S, Bhurgri M, Kayani N, Ahmed R, et al. Cancer profile of Hyderabad, Pakistan 1998-2002. Asian Pacific journal of cancer prevention : APJCP. 2005;6(4):474-80.

28. Bhurgri Y, Bhurgri A, Puri R, Ashraf S, Qidwai A, Ashraf K, et al. Rhabdomyosarcoma in Karachi 1998-2002. Asian Pacific journal of cancer prevention : APJCP. 2004;5(3):284-90.

29. Bhurgri Y, Bhurgri A, Rahim A, Bhutto K, Pinjani PK, Usman A, et al. The pattern of malignancies in Karachi (1995 to 1996). JPMA The Journal of the Pakistan Medical Association. 1999;49(7):157-61.

30. Bhurgri Y, Bhurgri A, Usman A, Pervez S, Kayani N, Bashir I, et al. Epidemiological review of head and neck cancers in Karachi. Asian Pacific journal of cancer prevention : APJCP. 2006;7(2):195-200.

31. Bhurgri Y, Bhurgri A, Usman A, Sheikh N, Faridi N, Malik J, et al. Patho-epidemiology of lung cancer in Karachi (1995-2002). Asian Pacific journal of cancer prevention : APJCP. 2006;7(1):60-4.

32. Bhurgri Y, Bhurgri H, Usman A, Faridi N, Malik J, Puri R, et al. Epidemiology of ocular malignancies in Karachi. Asian Pacific journal of cancer prevention : APJCP. 2003;4(4):352-7.

33. Bhurgri Y, Khan T, Kayani N, Ahmad R, Usman A, Bhurgri A, et al. Incidence and current trends of colorectal malignancies in an unscreened, low risk Pakistan population. Asian Pacific journal of cancer prevention : APJCP. 2011;12(3):703-8.

34. Bhurgri Y, Muzaffar S, Ahmed R, Ahmed N, Bhurgri H, Usman A, et al. Retinoblastoma in Karachi, Pakistan. Asian Pacific journal of cancer prevention : APJCP. 2004;5(2):159-63.

35. Bhurgri Y, Pervez S, Bhurgri A, Faridi N, Usman A, Kazi LA, et al. Increasing incidence of non-Hodgkin's lymphoma in Karachi, 1995-2002. Asian Pacific journal of cancer prevention : APJCP. 2005;6(3):364-9.

36. Cancer Profile of Larkana , Pakistan (2000-2002) %J Asian Pacific Journal of Cancer Prevention. 2006;7(4):518-21.

37. Bhurgri Y, Bhurgri A, Hasan SH. Comparability and Quality Control in Cancer Registration; Karachi (data monitoring 1995-2001). JPMA The Journal of the Pakistan Medical Association. 2002;52(7):301-7.

38. Bhurgri Y, Bhurgri A, Hasan SH, Usman A, Faridi N, Malik J, et al. Cancer patterns in Karachi division (1998-1999). JPMA The Journal of the Pakistan Medical Association. 2002;52(6):244-6.

39. Bhurgri Y, Bhurgri A, Hassan SH, Zaidi SH, Rahim A, Sankaranarayanan R, et al. Cancer incidence in Karachi, Pakistan: first results from Karachi Cancer Registry. International journal of cancer. 2000;85(3):325-9.

40. Bhurgri Y, Bhurgri H, Kayani N, Ahmad R, Usman A, Bhurgri A, et al. Trends and morphology of central nervous system malignancies in Karachi. Asian Pacific journal of cancer prevention : APJCP. 2011;12(8):2013-7.

41. Bhurgri Y, Bhurgri H, Pervez S, Kayani N, Usman A, Bashir I, et al. Epidemiology of soft tissue sarcomas in Karachi South, Pakistan (1995-7). Asian Pacific journal of cancer prevention : APJCP. 2008;9(4):709-14.

42. Bhurgri Y, Faridi N, Kazi LA, Ali SK, Bhurgri H, Usman A, et al. Cancer esophagus Karachi 1995-2002: epidemiology, risk factors and trends. JPMA The Journal of the Pakistan Medical Association. 2004;54(7):345-8.

43. Bhurgri Y, Kayani N, Faridi N, Pervez S, Usman A, Bhurgri H, et al. Patho-epidemiology of breast cancer in Karachi '1995-1997'. Asian Pacific journal of cancer prevention : APJCP. 2007;8(2):215-20.

44. Bhurgri Y, Kayani N, Pervez S, Ahmed R, Tahir I, Afif M, et al. Incidence and trends of prostate cancer in Karachi South, 1995-2002. Asian Pacific journal of cancer prevention : APJCP. 2009;10(1):45-8.

45. Bhurgri Y, Mazhar A, Bhurgri H, Usman A, Malik J, Bhurgri A, et al. Orbital embryonal rhabdomyosarcoma in Karachi (1998-2002). JPMA The Journal of the Pakistan Medical Association. 2004;54(11):561-5.

46. Bhurgri Y, Nazir K, Shaheen Y, Usman A, Faridi N, Bhurgri H, et al. Patho-epidemiology of cancer corpus uteri in Karachi South '1995-1997'. Asian Pacific journal of cancer prevention : APJCP. 2007;8(4):489-94.

47. Bhurgri Y, Nazir K, Shaheen Y, Usman A, Faridi N, Bhurgri H, et al. Patho-epidemiology of Cancer Cervix in Karachi South. Asian Pacific journal of cancer prevention : APJCP. 2007;8(3):357-62.

48. Bhurgri Y, Pervez S, Kayani N, Afif M, Tahir I, Nazir K, et al. Time trends in the incidence of cancer cervix in Karachi South, 1995-2002. Asian Pacific journal of cancer prevention : APJCP. 2008;9(3):533-6.

49. Bhurgri Y, Pervez S, Kayani N, Haider S, Ahmed R, Usman A, et al. Rising incidence of gastric malignancies in Karachi, 1995- 2002. Asian Pacific journal of cancer prevention : APJCP. 2009;10(1):41-4.

50. Bhurgri Y, Pervez S, Usman A, Khan JA, Bhurgri A, Kasi Q, et al. Cancer patterns in Quetta (1998-1999). JPMA The Journal of the Pakistan Medical Association. 2002;52(12):560-5.

51. Bhurgri Y, Rahim A, Bhutto K, Bhurgri A, Pinjani PK, Usman A, et al. Incidence of carcinoma of the oral cavity in Karachi--district south. JPMA The Journal of the Pakistan Medical Association. 1998;48(11):321-5.

52. Bhurgri Y, Shaheen Y, Kayani N, Nazir K, Ahmed R, Usman A, et al. Incidence, trends and morphology of ovarian cancer in Karachi (1995-2002). Asian Pacific journal of cancer prevention : APJCP. 2011;12(6):1567-71.

53. Bhurgri Y, Usman A, Bhurgri H, Faridi N, Bashir I, Bhurgri A, et al. Primary malignancies of bone and cartilage in Karachi. Asian Pacific journal of cancer prevention : APJCP. 2009;10(5):891-4.

54. Bhurgri Y, Bhurgri A, Hussainy AS, Faridi N, Bhurgri H, Usman A, et al. Incidence of cancer esophagus in Quetta and Karachi, Pakistan. Indian journal of gastroenterology : official journal of the Indian Society of Gastroenterology. 2003;22(5):170-2.

55. Chughtai N, Perveen K, Gillani SR, Abbas A, Chunara R, Manji AA, et al. National cervical cancer burden estimation through systematic review and analysis of publicly available data in Pakistan. BMC public health. 2023;23(1):834.

56. de Camargo Cancela M, Voti L, Guerra-Yi M, Chapuis F, Mazuir M, Curado MP. Oral cavity cancer in developed and in developing countries: population-based incidence. Head & neck. 2010;32(3):357-67.

57. Fadoo Z, Belgaumi A, Alam M, Azam I, Naqvi A. Pediatric Lymphoma: A 10-year Experience at a Tertiary Care Hospital in Pakistan. Journal of pediatric hematology/oncology. 2010;32:e14-8.

58. Hafeez S, Mahmood A, Khan R, Malkani N. Trends in Cancer Prevalence in Punjab, Pakistan: A Systematic Study from 2010 to 2016. Journal of Bioresource Management. 2020;7:68-78.

59. Hassan M, Butt ZA. Cancer research in Pakistan: Opportunities, challenges and the way forward. Journal of cancer policy. 2022;34:100358.

60. Dr. Aamir Hayat DZA, Dr. Pakiza Ishfaq. A DESCRIPTIVE TUMOR-BASED HISTOPATHOLOGICAL ANALYSIS ON MALIGNANCIES VARYING PATTERN. . Indo American Journal of Pharmaceutical Sciences 2018Jun15;05(06):5273–9

61. Hussain A, Ahmad S, Muhammad W, Kakakhel M, Matiullh M. Epidemiology of the breast cancer patients registered at Institute of Radiotherapy and Nuclear Medicine, Peshawar, Pakistan. European journal of cancer care. 2008;17:469-76.

62. Hussain Z GA, Qasmi SA. . Central Nervous System (CNS) Tumour Registry: A Single Neurosurgical Centre Experience of Four Years, <https://pakjns.org/index.php/pjns/article/view/66> (2016).

63. Ikram A, Pervez S, Khadim M, Sohaib M, Uddin H, Badar F, et al. National Cancer Registry of Pakistan: First Comprehensive Report of Cancer Statistics 2015-2019. Journal of the College of Physicians and Surgeons--Pakistan : JCPSP. 2023;33:625-32.

64. Jahangir S, Loya A, Mushtaq S, Akhter N, Hashmi AA. CD117/c-KIT Expression in Phyllodes Tumor of the Breast and Its Correlation With Morphology and Clinical Outcome. Cureus. 2021;13(5):e14914.

65. Jamal S, Atique M, Khadim MT. Changing pattern of malignancies: analysis of histopathology based tumour registry data and comparison of three decades at Armed Forces Institute of Pathology, Rawalpindi, Pakistan. JPMA The Journal of the Pakistan Medical Association. 2014;64(1):24-7.

66. Jamal S, Khadim MT, Din HU, Akhtar F, Khan AA, Parveen B, et al. SPECTRUM OF MALIGNANT TUMOURS IN ADOLESCENCE AND YOUNG ADULTS: AN ANALYSIS OF 1873 CASES. Pakistan Armed Forces Medical Journal. 2020;70(1):27-31.

67. Jamal S, Khadim MT, Din HU, Asif M, Ahmad R, Rashid F. Childhood cancers: Analysis of 1279 cases at Armed Forces Institute of Pathology, Rawalpindi, Pakistan. JPMA The Journal of the Pakistan Medical Association. 2020;70(6):1009-12.

68. Jamal S, Mamoon N, Mushtaq S, Luqman M. Analysis of gastrointestinal malignancies at the Armed Forces Institute of Pathology (AFIP), Rawalpindi, Pakistan. Asian Pacific journal of cancer prevention : APJCP. 2005;6(4):497-500.

69. Jamal S, Mamoon N, Mushtaq S, Luqman M. Pattern of childhood malignancies: study of 922 cases at Armed Forces Institute of Pathology (AFIP), Rawalpindi, Pakistan. Asian Pacific journal of cancer prevention : APJCP. 2006;7(3):420-2.

70. Jamal S, Mamoon N, Mushtaq S, Luqman M, Moghal S. The pattern of gynecological malignancies in 968 cases from Pakistan. Annals of Saudi medicine. 2006;26(5):382-4.

71. Jamal S, Moghal S, Mamoon N, Mushtaq S, Luqman M, Anwar M. The pattern of malignant tumours: tumour registry data analysis, AFIP, Rawalpindi, Pakistan (1992-2001). JPMA The Journal of the Pakistan Medical Association. 2006;56(8):359-62.

72. Jamal S MN, Mushtaq S, Luqman M. . Pattern of central nervous system (CNS) tumor: a study of 430 cases. . Pak J Pathol 2005; 16: 106-109.

73. Janjua OS, Ahmed W, Qureshi SM, Khan TS, Ahmed A, Alamgir W. Assessment of margins in resection specimens for head and neck malignancies. Journal of the College of Physicians and Surgeons--Pakistan : JCPSP. 2013;23(4):265-8.

74. Javed A. Progress of Oncology in Pakistan. Indian Journal of Medical and Paediatric Oncology. 2006;27:54-9.

75. Kayani BF, Mamoon N, Jamal S, Ahmad SA, Khadim MT, Mushtaq S. Spectrum of primary non-cutaneous malignant melanomas in northern Pakistan. Asian Pacific journal of cancer prevention : APJCP. 2011;12(1):283-4.

76. Khadim MT, Jamal S, Ali Z, Akhtar F, Atique M, Sarfraz T, et al. Diagnostic challenges and role of immunohistochemistry in metastatic liver disease. Asian Pacific journal of cancer prevention : APJCP. 2011;12(2):373-6.

77. Mahmood S FR, Yousaf A, Quader AU, Asif H, Atif A, et al. . ANNUAL CANCER REGISTRY REPORT-2017, OF THE SHAUKAT KHANUM MEMORIAL CANCER HOSPITAL & RESEARCH CENTER, PAKISTAN. 2017.

78. Mamoon N, Hassan U, Mushtaq S. Breast carcinoma in young women aged 30 or less in Northern Pakistan - the Armed Forces Institute of Pathology experience. Asian Pacific journal of cancer prevention : APJCP. 2009;10(6):1079-82.

79. Masood A, Masood K, Hussain M, Ali W, Riaz M, Alauddin Z, et al. Thirty Years Cancer Incidence Data for Lahore, Pakistan: Trends and Patterns 1984-2014. Asian Pacific journal of cancer prevention : APJCP. 2018;19(3):709-17.

80. Munawar K, Qazi R, Sheikh HS. Utilisation Patterns and Treatment Outcomes of EGFR-Tyrosine Kinase Inhibitors in EGFR-mutant Advanced Lung Carcinoma in the Pakistani-Asian Population: A Real-world Data Study. Journal of cancer & allied specialties. 2023;9(1):491.

81. Mushtaq S, Akhtar N, Jamal S, Mamoon N, Khadim T, Sarfaraz T, et al. Malignant lymphomas in Pakistan according to the WHO classification of lymphoid neoplasms. Asian Pacific journal of cancer prevention : APJCP. 2008;9(2):229-32.

82. Nawaz M, Bilal M, Asgher M. Prevalence of Lymphoma Cancer in Punjab, Pakistan. International Journal of Applied Sciences and Biotechnology. 2015;3.

83. Pervez S. Non-Hodgkin Lymphoma (NHL) in Pakistan. International journal of molecular and cellular medicine. 2012;1(1):62-3.

84. Pervez S. Karachi Cancer Registry (KCR): Age-Standardized Incidence Rate by Age-Group and Gender in a Mega City of Pakistan. 2020.

85. Pervez S, Jabbar AA, Haider G, Qureshi MA, Ashraf S, Lateef F, et al. Karachi Cancer Registry (KCR): Consolidated Data of 5-years 2017-2021. Journal of the College of Physicians and Surgeons--Pakistan : JCPSP. 2023;33(5):560-5.

86. Qureshi MA, Khan S, Sharafat S, Quraishy MS. Common Cancers in Karachi, Pakistan: 2010-2019 Cancer Data from the Dow Cancer Registry. Pakistan journal of medical sciences. 2020;36(7):1572-8.

87. Qureshi MA, Mirza T, Khan S, Sikandar B, Zahid M, Aftab M, et al. Cancer patterns in Karachi (all districts), Pakistan: First results (2010-2015) from a Pathology based cancer registry of the largest government-run diagnostic and reference center of Karachi. Cancer epidemiology. 2016;44:114-22.

88. Qureshi MA, Syed SA, Sharafat S. Lip and oral cavity cancers (C00-C06) from a mega city of Pakistan: Ten-year data from the Dow Cancer Registry. Journal of Taibah University Medical Sciences. 2021;16(4):624-7.

89. Rahman B, Mamoon N, Jamal S, Zaib N, Luqman M, Mushtaq S, et al. Malignant tumors of the minor salivary glands in northern Pakistan: a clinicopathological study. Hematology/oncology and stem cell therapy. 2008;1(2):90-3.

90. Sarwar CM, Siddiqui N, Khokhar RA, Badar F. Epithelial ovarian cancer at a cancer hospital in a developing country. Asian Pacific journal of cancer prevention : APJCP. 2006;7(4):595-8.

91. Shafqat Ali S, Syed Akbar A, Muhammad Ehteram Ul H, Bushra A, Talha Ahmed Q, Amna K. To assess the aggressiveness of oral squamous cell carcinoma in the young population. Journal of the Pakistan Medical Association. 2022;72(10):1937-41.

92. Sheikh HS, Munawar K, Sheikh F, Qamar MFU. Lung Cancer in Pakistan. Journal of thoracic oncology : official publication of the International Association for the Study of Lung Cancer. 2022;17(5):602-7.

93. Shamim F, Khan AA, Khan FA. First-Pass Success of Tracheal Intubation With Videolaryngoscopy in Head and Neck Cancer Patients: A Registry-Based Retrospective Cohort Study. Cureus. 2021;13(12):e20857.

94. Siddiqui N, Ayub B, Badar F, Zaidi A. Hodgkin's lymphoma in Pakistan: a clinico-epidemiological study of 658 cases at a cancer center in Lahore. Asian Pacific journal of cancer prevention : APJCP. 2006;7(4):651-5.

95. Sultan S, Irfan SM, Ali N, Nawaz N. Institutional-based tumor registry of hematopoietic malignancies: A 4 years' preliminary report from Karachi. Journal of laboratory physicians. 2018;10(2):168-72.

96. Tufail M, Wu C. Exploring the Burden of Cancer in Pakistan: An Analysis of 2019 Data. Journal of epidemiology and global health. 2023;13(2):333-43.

97. Umer M, Askari R, Baz S. Use of fresh parental fibular allograft for reconstruction of skeletal defects after limb salvage surgery. JPMA The Journal of the Pakistan Medical Association. 2014;64(12 Suppl 2):S151-3.

98. Umer M, Saeed J, Shamsi Z, Tariq MU. Treatment and outcomes of soft tissue sarcoma of groin, hip and thigh: a retrospective review from a tertiary care hospital. JPMA The Journal of the Pakistan Medical Association. 2021;71(Suppl 5)(8):S75-s8.

99. Wickramasinghe DP, Samarasekera DN. Incidence of esophageal cancer in Sri Lanka: Analysis of cancer registry data and comparison with other South Asian populations. Asia-Pacific journal of clinical oncology. 2017;13(5):e271-e7.

100. Yusuf A. Cancer Care in Pakistan. Japanese Journal of Clinical Oncology. 2013;43(8):771-5.

101. Zeb A, Rasool A, Nasreen S. Occupation and cancer incidence in District Dir (NWFP), Pakistan, 2000-2004. Asian Pacific journal of cancer prevention : APJCP. 2006;7(3):483-4.

102. Zeb A, Rasool A, Nasreen S. Cancer incidence in the districts of Dir (North West Frontier Province), Pakistan: a preliminary study. Journal of the Chinese Medical Association : JCMA. 2008;71(2):62-5.
